# Silica: An efficient catalyst for one-pot regioselective synthesis of dithioethers

**DOI:** 10.3762/bjoc.10.5

**Published:** 2014-01-07

**Authors:** Samir Kundu, Babli Roy, Basudeb Basu

**Affiliations:** 1 Department of Chemistry, North Bengal University, Darjeeling 734013, India, Fax: +91 353 2699001

**Keywords:** allyl halide, dithioether, silica gel, tandem reactions, thiol

## Abstract

The development of a silica-promoted highly selective synthesis of 1,2 or 1,3-dithioethers via solvent-free one-pot tandem reactions of an allyl bromide with excess thiol at room temperature is described. The choice of silica gel, either pre-calcined or moistened with water, exhibited notable regioselectivity in the formation of dithioethers. Plausible mechanistic routes were explored and postulated.

## Introduction

Organosulfur compounds are important building blocks for the synthesis of various biologically active molecules [1–3]. Versatile applications of organosulfur compounds are known in fields such as the pharmaceutical, the polymer, the pesticide and the food-processing industry [4–8]. For example, organosulfur compounds in garlic are often used in food-processing industries as flavouring and preservative agents and are also used as herbal medicine [4]. Dithioethers are commonly employed as ligands in preparing metal-coordination complexes and also as spacers in metal-organic frameworks [9–14]. For example, vicinal dithioether-based zirconium and titanium complexes have been used for alkene polymerization and hydroamination [15–18]. Chiral dithioethers have been prepared and their iridium complexes have been employed in asymmetric hydrogenation [18]. Vicinal dithioethers are generally synthesised either by the metal-catalyzed addition of disulfides to alkenes [19–20] or by the traditional nucleophilic substitution of 1,2-dihalides with suitable thiols/thiolates [21–22]. They are also prepared by consecutive hydrothiolation of alkynes, both under nucleophilic and radical-induced conditions [22–23]. On the other hand, 1,3-dithioethers can be prepared by the nucleophilic substitution of compounds bearing suitable leaving groups at 1,3-positions of alkyl chains [21]. Because of their versatile applications, a great number of procedures have been developed to synthesize bis(thioethers) with varying degrees of success and a variety of limitations [19–31].

**Scheme 1:**
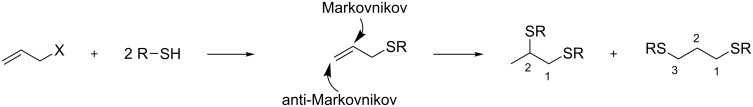
Sequential substitution-addition reactions of thiols with allyl halides leading to the formation of 1,2 or 1,3-dithioethers.

Over the last decade, organic synthesis has taken a major turn towards developing reaction conditions that are environmentally friendly and sustainable [32–36]. Mesoporous inorganic oxides, which often facilitate various organic reactions, are considered suitable to promote eco-friendly chemical processes [36]. Organic reactions with a high selectivity under eco-friendly and sustainable conditions are attractive features in terms of the concepts of Green chemistry. Previously, we have developed silica-promoted facile and highly selective methods for N and S-alkylations/acylation from amines or thiols, respectively [37–38]. An equimolar mixture of a benzenethiol and allyl bromide on treatment with silica afforded allyl(phenyl)sulfane in excellent yield. Since alkenes are also known to undergo ‘click’ addition with thiols [39–40], excess use of thiols could effectively produce dithioethers, and based on a regioselective addition one could achieve either vicinal or 1,3-dithioethers in one-pot consecutive substitution–hydrothiolation processes (Scheme 1). Although both reactions are well-known, a search in the literature surprisingly revealed no general one-pot protocols for the preparation of dithioethers from allylic substrates. Recently, Banerjee and co-workers reported on the simple synthesis of thioethers by silica NPs, where a single example of a reaction of an allyl bromide and excess benzenethiol was studied [41–42]. The reaction was carried out in the presence of silica NPs and water, and they isolated 1,3-dithioether by an anti-Markovnikov addition. However, there is no report on the metal-free hydrothiolation of allylic substrates in a Markovnikov fashion to afford 1,2-dithioethers in one-pot reactions. In this paper, we wish to report our investigations on the reaction of allyl halides with excess thiols promoted by silica gel, which finally constitutes distinct protocols for one-pot, solvent-free substitution and regioselective additions to produce either 1,2 or 1,3-dithioethers.

## Results and Discussion

Following our previous experience [37–38], we first attempted the magnetic stirring of a mixture of allyl bromide and benzenethiol in a 1:2.5 ratio by using pre-calcined silica gel at room temperature that indeed led to the formation of 1,2-dithioether in 91% yield. On the other hand, if silica gel moistened with a few drops of water was used for the same reaction, the regioselective anti-Markovnikov addition product, i.e., 1,3-dithioether, (1-(3-(phenylthio)propylthio)benzene) was obtained in 83% yield. In both cases, a minimal amount of diphenyldisulfide (5–10%) was formed [43–44], which was easily separable from the reaction mixture by column chromatography. Since the choice of silica led to the production of highly regioselective products, we wanted to optimize both conditions to establish them as general protocols. Table 1 shows the optimization of the reactions of different allylic substrates with benzenethiol. Silica gel (directly from the container, commercially available) was used either pre-activated by heating at 100 °C under vacuum for 1 h and then cooled under vacuum for use under conditions A or moist with water (0.1 mL water for 0.5 g of silica) for use under conditions B. It was observed that allyl bromide or allyl iodide underwent sequential substitution–addition reactions entirely regioselectively with comparable yields (Table 1, entries 1–5), whereas allyl chloride showed varying results under conditions A or B, and allyl acetate did not undergo any desired reaction, but merely produced the disulfide from oxidative dimerization of the thiol (Table 1, entries 6–8). Allyl tosylate, however, produced the desired thioethers in a regioselective manner, but with relatively low yields (Table 1, entries 9 and 10). Interestingly, allylphenylsulfane or allyl phenyl ether entirely followed an anti-Markovnikov addition, under both conditions, A and B (Table 1, entries 11–14).

**Table 1:** Optimization of one-pot sequential substitution–hydrothiolation of allylic substrate with excess benzenethiol over silica at room temperature.

Entry	CH_2_=CH-CH_2_-X	Conditions^a^	Time (h)	Product^b^/Yield^c^ (%)

1	X = Br	A	6	1,2-dithioether/77
2	X = Br	A	11	1,2-dithioether/91
3	X = Br	B	20	1,3-dithioether/83
4	X = I	A	12	1,2-dithioether/89
5	X = I	B	20	1,3-dithioether/85
6	X = Cl	A	15	1,2-dithioether/57
7	X = Cl	B	30	diphenyldisulfide/83
8	X = OAc	A	24	diphenyldisulfide/90
9	X = OTs	A	8	1,2-dithioether/75
10	X = OTs	B	22	1,3-dithioether/68
11^d^	X = SPh	A	5	1,3-dithioether/83
12^d^	X = SPh	B	12	1,3-dithioether/80
13^d^	X = OPh	A	6	3-phenoxythioether/89
14^d^	X = OPh	B	14	3-phenoxythioether/82
15	X = Br	Neat mixture	20	no dithioether is formed

^a^Conditions A: allylic compound and PhSH (1:2.5 mmol) over pre-calcined dry silica gel (0.5 g); conditions B: allylic compound and PhSH (1:2.5 mmol) over moistened silica gel (0.5 g). ^b^In each case 5–10% diphenyldisulfide was formed except in entries 6–8. ^c^Yield refers to isolated pure product and no other constitutional isomer was detected. ^d^Thiol (1.2 mmol) was used for entries 11–13.

With the two distinct conditions, we examined the scope of these one-pot tandem reactions of allyl bromide with a variety of thiols under both conditions. The results are presented in Table 2. Arylthiols bearing different functional groups like CH_3_, OCH_3_, Cl or F were reacted with allyl bromide in the presence of pre-calcined and dry silica affording good to excellent yields of the corresponding 1,2-dithioethers (Table 2, entries 1, 3, 5, 7, 9, 11 and 17). 2-Naphthylthiol also underwent a similar regioselective Markovnikov addition, resulting in the corresponding 1,2-dithioether in 82% yield (Table 2, entry 18). Extending the protocol to aliphatic thiols, such as *n*-pentylthiol and cyclohexylthiol also afforded regioselective dithioether in good yields (Table 2, entries 13 and 15). In all the cases, we observed 100% Markovnikov addition products and no anti-Markovnikov products were detected. We now turned our attention to the other conditions B – the use of moist silica gel. Again, a variety of aromatic thiols, including those that were used for the conditions A, were employed to react with allyl bromide in the presence of silica moist with a few drops of water, and we isolated entirely regioselective 1,3-dithioethers (Table 2, entries 2, 4. 6, 8, 10 and 12). The same selectivity was observed in the reaction of aliphatic thiols (acyclic or alicyclic), viz. *n*-pentane-1-thiol and cyclohexanethiol, with allyl bromide to afford the corresponding 1,3-dithioethers in 71% and 67% yield, respectively (Table 2, entries 14 and 16). In these cases, we did not detect any Markovnikov addition products. Thus, moistened silica gel turns out to be effective for sequential substitution reactions, and entirely anti-Markovnikov addition, while pre-calcined dry silica gel could efficiently give rise to only Markovnikov addition products. The reactions over dry silica gel appear to be faster than the procedure using moist silica. Moreover, the 1,2-dithioethers are formed in slightly better yields than the corresponding 1,3-analogues. We also experienced that aromatic thiols, under both conditions A and B, give better yields than aliphatic thiols.

**Table 2:** Regioselective one-pot synthesis of 1,2 and 1,3-dithioethers using dry (pre-calcined) or moistened silica gel at room temperature.

Entry	Thiol	Conditions^a^	Time (h)	Product	Yield^b^ (%)

1	C_6_H_5_-SH	A	10	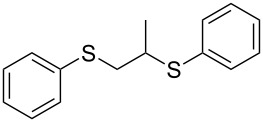	91
2	C_6_H_5_-SH	B	22	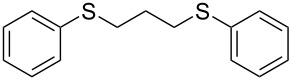	81
3	4-(H_3_C)C_6_H_4_-SH	A	6	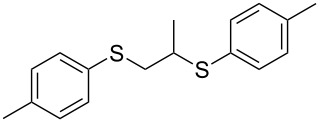	87
4	4-(H_3_C)C_6_H_4_-SH	B	20	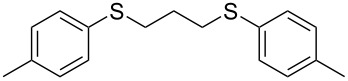	78
5	4-(H_3_CO)C_6_H_4_-SH	A	6.5	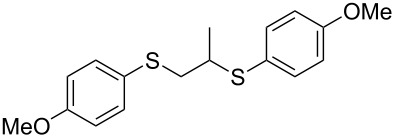	78
6	4-(H_3_CO)C_6_H_4_-SH	B	18	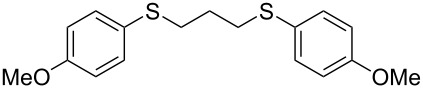	76
7	4-(Cl)C_6_H_4_-SH	A	6	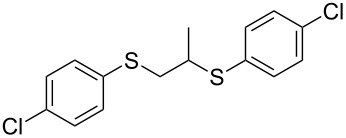	83
8	4-(Cl)C_6_H_4_-SH	B	15	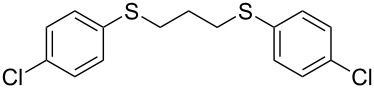	87
9	4-(F)C_6_H_4_-SH	A	8	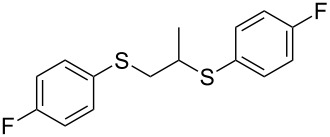	80
10	4-(F)C_6_H_4_-SH	B	16	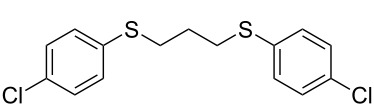	84
11	2,6-(CH_3_)_2_)C_6_H_3_-SH	A	8	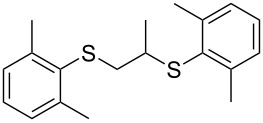	74
12	2,6-(CH_3_)_2_)C_6_H_3_-SH	B	20	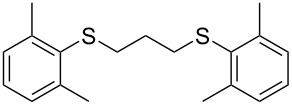	77
13	*n*-C_5_H_11_-SH	A	9	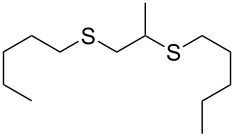	67
14	*n*-C_5_H_11_-SH	B	16	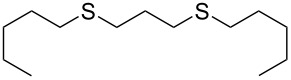	71
15	Cy-SH	A	10	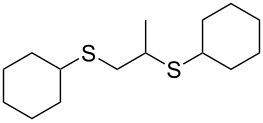	65
16	Cy-SH	B	18	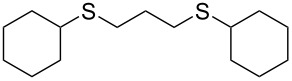	67
17	2-(H_3_C)C_6_H_4_-SH	A	7	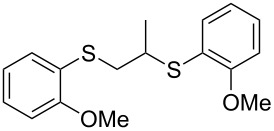	71
18	2-C_10_H_7_-SH	A	9	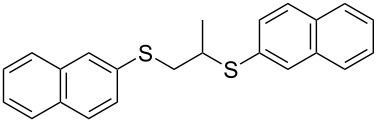	82
19^c^	C_6_H_5_-SH	B	22	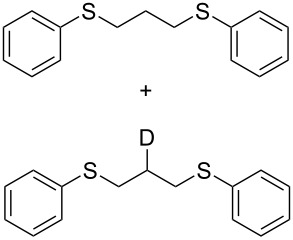	
20	C_6_H_5_-SH	A^d^	15	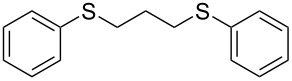	82

^a^Conditions A: allylic compound and PhSH (1:2.5 mmol) over pre-calcined dry silica gel (0.5 g); conditions B: allylic compound and PhSH (1:2.5 mmol) over moist silica gel (0.5 g). ^b^Yield refers to isolated pure product; in each case 5–10% diphenyldisulfide was formed and isolated. ^c^D_2_O (0.5 mL for 0.5 g silica gel) was used instead of H_2_O. ^d^Mixture of silica and sodium silicate (1:1 w/w; 0.5 g for 1 mmol of allyl bromide) was used after drying under vacuum.

**Scheme 2:**
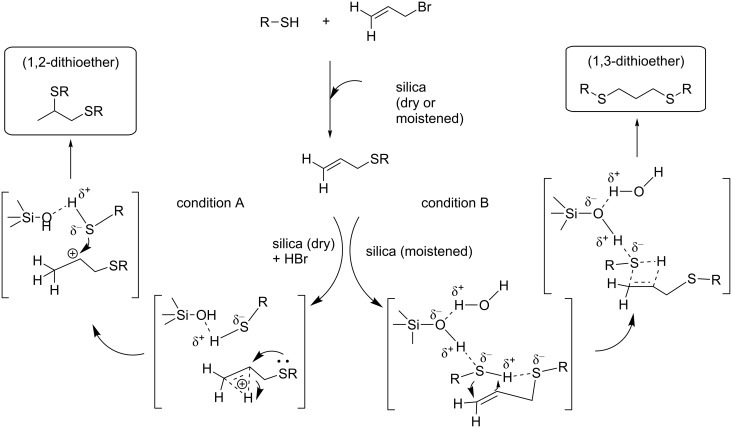
Plausible mechanisms for the regioselective formation of vicinal and 1,3-dithioethers by using dry or moistened silica gel.

We assume that the nature of the silica surface and its possible interactions with thiols is responsible for the notable regioselectivity in the hydrothiolation of allylsulfane. It is known that amorphous or mesoporous silica consists of silanol groups and siloxane bridges that determine its surface properties, and the concentration of these OH groups depends mostly on the actual process of calcinations [45–47]. Based on Zhuravlev’s physicochemical model of silica surface [45], it may be presumed that the moistened silica surface is covered with a single layer or multilayer of adsorbed water, which might disappear during the calcination process. Since allylphenylsulfane on hydrothiolation affords the anti-Markovnikov product under both conditions A and B (Table 1, entries 11 and 12), we presume that there might be an influence of the generated acid in the first step under dry conditions A. In the absence of water, the generated HBr in the first step might activate the double bond and subsequent assistance by the neighbouring sulfur atom coupled with the stability of the secondary carbocation lead to the Markovnikov addition resulting in the exclusive formation of 1,2-dithioether (Scheme 2, conditions A). On the other hand, the moist silica consisting of a single layer or multilayered adsorbed water promotes thiols to bind with allylsulfane, and the subsequent addition takes place in an anti-Markovnikov approach (Scheme 2, conditions B).

In order to find evidence for the role of silica adsorbed water, we conducted the following experiments: (i) the reaction was carried out under conditions A in the presence of an exogenous base (sodium silicate; see Experimental), which leads to the formation of anti-Markovnikov product only (1,3-dithioether) (Table 2, entry 20); (ii) reactions under conditions B with varying quantities of H_2_O (0.5 mL and 1.0 mL) did not exhibit any significant changes producing only 1,3-dithioethers in quantities; (iii) dry HCl gas was passed through pre-calcined silica and was used for the hydrothiolation of allylphenylsulfane, exclusively yielding an Markovnikov addition product (1,2-dithioether); (iv) an experiment was carried out with silica moistened with D_2_O (Table 2, entry 19), which afforded a mixture of 1,3-bis(phenylthio)propane and [2-D]1,3-dithioether as seen from the ^1^H NMR spectrum of the mixture and calculated to be in the ratio of 1:3.9. In the ^13^C NMR spectrum, the deuterated carbon appeared as a triplet at δ 27.96, *J* = 20 Hz (see Supporting Information File 1). This observation supports that conditions B might occur through an initial thiol proton exchange with D_2_O (PhS–H → PhS–D).

## Conclusion

We have demonstrated that the choice of silica gel, either dry or moistened, could lead to highly selective pathways for the preparation of different dithioethers. The sequential reactions in one-pot protocols are robust, neutral, metal-free and notably selective with a broad range of substrates. The diverse reactivity of silica gel in the formation of vicinal or 1,3-dithioethers might not only spur the adaptation of existing procedures for selective dithioether preparation but also attract novel applications. Further utilizations of this diverse reactivity are currently being explored in our laboratory.

## Experimental

All chemicals were purchased from commercial suppliers and used without further purification. IR spectra were recorded on an FTIR spectrophotometer (8300 Shimadzu) using Nujol mulling for liquid compounds and KBr pellets for solid compounds. NMR spectra were recorded on a Varian AV-300 spectrometer with CDCl_3_ as a solvent. Chemical shifts (δ) are reported in ppm and referenced to TMS for ^1^H NMR spectra and residual solvent signals for ^13^C NMR spectra as internal standards. Coupling constants (*J*) are reported in Hertz (Hz). Standard abbreviations indicating multiplicity were used as follows: s = singlet, d = doublet, t = triplet, q = quartet, qnt = quintet, m = multiplet. Melting points were determined by heating in an open capillary tube. High resolution mass spectra (HRMS) were performed in a Micromass Q-TOF Spectrometer under ESI (positive mode) by the services at the Indian Association for the Cultivation of Science, Kolkata.

**Calcination**: Commercially available silica gel (Merck, India; Grade: TLC; HF_254_) was heated under vacuum at 100 °C for 1 h, cooled, and then be used for the reaction or stored in a glass-stoppered flask for at least two weeks.

**Moistened silica**: Commercially available silica gel (Merck, India; Grade: TLC; HF_254_) was mixed with water and used for the reactions. For column chromatography: silica (60–120 µm) (Thomas Baker, India), and for TLC, Merck plates coated with silica gel 60, F_254_ were used.

### General procedure for Table 2 (route A or B)

Route A: A mixture of allyl bromide (1 mmol) and thiol (2.5 mmol) was mixed with pre-calcined dry silica gel (Table 2, for entries 1, 3, 5, 7, 9, 11, 13, 15, 17 and 18)

Route B: A mixture of allyl bromide (1 mmol) and thiol (2.5 mmol) was mixed with silica gel (0.5 g), moistened with two drops of water, (Table 2, for entries 2, 4, 6, 8, 10, 12, 14 and 16), and stirred magnetically by using a spin bar for the respective times listed in Table 2. The reaction was monitored by TLC. After completion the product was purified by column chromatography over silica gel. Elution with light petroleum or mixtures of ethyl acetate/light petroleum (see Supporting Information File 1) furnished the desired dithioether. All products were characterized by IR, ^1^H NMR, ^13^C NMR and HRMS data.

### Procedure for the reaction using a mixture of silica and sodium silicate under conditions A (Table 2, entry 20)

Equal quantities of silica gel and sodium silicate (1 g each) were mixed, dried under vacuum at 100 °C for 1 h, cooled, and used for the reaction. The mixture (500 mg) was stirred in water (10 mL) and its pH was measured to be 12.7. A mixture of allyl bromide (1 mmol) and benzene thiol (2.5 mmol) was thoroughly mixed with the mixture of dry silica gel and sodium silicate (500 mg), and the solid reaction mixture stirred for 15 h at room temperature. After the reaction, the product was purified by column chromatography (82% yield) and characterized as 1-(3-(phenylthio)propylthio)benzene (1,3-dithioether).

## Supporting Information

Supporting information features FTIR, ^1^H NMR, ^13^C NMR and HRMS data for 1,2 and 1,3-dithioethers (Table 2, entries 1–19) and ^1^H NMR and ^13^C NMR spectra for compounds listed in Table 2, entries 1–19.

File 1 Characterization data for compounds listed in Table 2, entries 1–19.

File 2 ^1^H NMR and ^13^C NMR spectra for compounds listed in Table 2, entries 1–19.
