# Supplementary material for: Silica: An efficient catalyst for one-pot regioselective synthesis of dithioethers
Source: Beilstein J Org Chem. 2014 Jan 7;10:26–33. doi: 10.3762/bjoc.10.5 (PMC3896229; doi:10.3762/bjoc.10.5)
Supplement: File 1 — Characterization data for compounds listed in Table 2, entries 1–19. [file Beilstein_J_Org_Chem-10-26-s001.pdf]

Supporting Information

for

Silica: An efficient catalyst for one-pot regioselective synthesis of dithioethers

Samir Kundu[1], Babli Roy[1] and Basudeb Basu*[1]

Address: [1]Department of Chemistry, North Bengal University, Darjeeling 734013, India,

Fax: +91 353 2699001

Email: Basudeb Basu* - basu_nbu@hotmail.com

*Corresponding author

Characterization data for the compounds listed in Table 2, entries 1–19

Characterization data for the compounds listed in Table 2 (entries 1–19)

1-(1-(Phenylthio)propan-2-ylthio)benzene [1],

Entry 1; Obtained as pale yellow liquid (elution with light petroleum).

IR (Nujol) 1581.5, 740.6, 690.5 cm^{-1}; ^1H NMR ($CDCl_3$, 300 MHz): δ 1.40 (d, *J* = 6.2 Hz, 3H, CH_3), 2.72-2.80 (m, 1H, CH), 3.19-3.29 (m, 2H, CH_2), 7.11-7.33 (m, 10H, ArH); ^{13}C NMR ($CDCl_3$, 75 MHz): δ 19.2, 40.4, 42.3, 126.1, 127.2, 128.8, 129.5, 132.5, 133.9, 135.6; HRMS (ESI) calcd for $C_{15}H_{16}KS_2$ 299.0330; found 299.0331.

1-(3-(Phenylthio)propylthio)benzene [2],

Entry 2; Obtained as colourless liquid (elution with light petroleum).

IR (Nujol) 690.5, 736.8 cm^{-1}; ^1H NMR ($CDCl_3$, 300 MHz): δ 1.93 (quintet, *J* = 6.9 Hz, 2H, CH_2), 3.02 (t, *J* = 6.9 Hz, 4H, $2CH_2$), 7.12-7.17 (m, 2H, ArH), 7.21-7.31 (m, 8H, ArH); ^{13}C NMR ($CDCl_3$, 75 MHz): δ 28.2, 32.3, 126.0, 128.8, 129.2, 135.9; HRMS (ESI) calcd for $C_{15}H_{16}KS_2$ 299.0330; found 299.0331.

1-(2-(*p*-Tolylthio)propylthio)-4-methylbenzene,

Entry 3; Obtained as pale yellow liquid (elution with light petroleum).

IR (Nujol) 1492.8, 802.3, 721.3 cm^{-1}; ^1H NMR ($CDCl_3$, 300 MHz): δ 1.36 (d, *J* = 6.9 Hz, 3H, CH_3), 2.27 (s, 3H, $ArCH_3$), 2.30 (s, 3H, $ArCH_3$), 2.70-2.74 (m, 1H, CH), 3.17-3.24 (m, 2H, CH_2), 6.98-7.22 (m, 6H, ArH), 7.21 (d, *J* = 8.1 Hz, 2H, ArH); ^{13}C NMR ($CDCl_3$, 75 MHz): δ 19.2, 20.9, 21.0, 41.0, 42.5, 129.5, 130.0, 130.1, 132.0, 133.0, 136.1, 137.2; HRMS (ESI) calcd for $C_{17}H_{20}KS_2$ 327.0643; found 327.0645.

1-(3-(*p*-Tolylthio)propylthio)-4-methylbenzene,

Entry 4; Obtained as colourless liquid (elution with light petroleum).

IR (Nujol) 1492.8, 1091.6, 1018.3, 802.3, 721.3, 505.3 cm^{-1}; ^1H NMR ($CDCl_3$, 300 MHz): δ 1.83 (qnt, *J* = 6.9 Hz, 2H, CH_2), 2.26 (s, 6H, $2ArCH_3$), 2.92 (t, *J* = 6.9 Hz, 4H, $2CH_2$), 7.02 (d, *J* = 7.8 Hz, 4H, ArH), 7.17 (d, *J* = 8.4 Hz, 4H, ArH); ^{13}C NMR ($CDCl_3$, 75 MHz): δ 21.0, 28.4, 33.1, 129.6, 130.2, 132.1, 136.2; HRMS (ESI) calcd for $C_{17}H_{20}KS_2$ 327.0643; found 327.0644.

1-(1-(4-Methoxyphenylthio)propan-2-ylthio)-4-methoxybenzene,

Entry 5; Obtained as colourless liquid (elution with 3% ethyl acetate - light petroleum).

IR (Nujol) 1593.1, 1492.8, 1284.5, 1245.9, 1176.5, 1099.3, 1033.8, 1006.8, 825.5, 640.0, 524.6 cm^{-1}; ^1H NMR ($CDCl_3$, 300 MHz): δ 1.35 (d, *J* = 6.6 Hz, 3H, CH_3), 2.63-2.71 (m, 1H, CH), 3.00-3.16 (m, 2H, CH_2), 3.78 (s, 3H, OCH_3), 3.79 (s, 3H, OCH_3), 6.75-6.82 (m, 4H, ArH), 7.17-7.22 (m, 2H, ArH), 7.25-7.30 (m, 2H, ArH); ^{13}C NMR ($CDCl_3$, 75 MHz): δ 19.2, 42.2, 43.2, 55.2, 114.3, 114.5, 123.9, 125.9, 132.9, 135.6, 158.8, 159.5; HRMS (ESI) calcd for $C_{17}H_{20}O_2KS_2$ 359.0542; found 359.0543.

1-(3-(4-Methoxyphenylthio)propylthio)-4-methoxybenzene,
Entry 6; Obtained as colourless liquid (elution with 3% ethyl acetate - light petroleum).
IR (Nujol) 1593.1, 1492.8, 1284.5, 1242.1, 1176.5, 1033.8, 825.5, 620.0, 525.0 cm^{-1}; 1H NMR ($CDCl_3$, 300 MHz): δ 1.80 (qnt, *J* = 6.9 Hz, 2H,CH_2), 2.90 (t, *J*= 6.9 Hz, 4H, $2CH_2$), 3.78 (s, 6H, $2OCH_3$), 6.78-6.83 (m, 4H, ArH), 7.25-7.32 (m, 4H,ArH); ^{13}C NMR ($CDCl_3$, 75 MHz): δ 28.5, 34.4, 55.2, 114.5, 125.9, 133.4, 158.9; HRMS (ESI) calcd for $C_{17}H_{20}O_2KS_2$ 359.0542; found 359.0540.

1-(2-(4-Chlorophenylthio)propylthio)-4-chlorobenzene,
Entry 7; Obtained as pale yellow liquid (elution with light petroleum).
IR (Nujol) 1176.5, 1095.5, 1010.6, 817.8, 744.5, 550.0, 493.7 cm^{-1}; ^1H NMR ($CDCl_3$, 300 MHz): δ 1.37 (d, *J* = 6.6 Hz, 3H, CH_3), 2.71-2.79 (m, 1H, CH), 3.12-3.19 (m, 2H, CH_2), 7.10 (d, *J* = 8.4 Hz, 2H, ArH), 7.17 (d, *J* = 8.4 Hz, 2H, ArH), 7.21 (s, 4H, ArH); ^{13}C NMR ($CDCl_3$, 75 MHz): δ 19.2, 40.8, 42.7, 128.9, 129.0, 131.0, 132.3, 133.5, 133.8, 134.0; HRMS (ESI) calcd for $C_{15}H_{14}Cl_2KS_2$ 366.9551; found 366.9557.

1-(3-(4-Chlorophenylthio)propylthio)-4-chlorobenzene,
Entry 8; Obtained as colourless liquid (elution with light petroleum).
IR (Nujol) 1249.8, 1095.5, 1010.6, 813.9, 744.5, 540.0, 489.9 cm^{-1}; ^1H NMR ($CDCl_3$, 300 MHz): δ 1.89 (quintet, *J* = 6.9 Hz, 2H, CH_2), 2.99 (t, *J* = 6.9 Hz, 4H, $2CH_2$), 7.22 (s, 8H, ArH); ^{13}C NMR ($CDCl_3$, 75 MHz): δ 28.0, 32.5, 129.0, 130.7, 132.1, 134.3; HRMS (ESI) calcd for $C_{15}H_{14}Cl_2KS_2$ 366.9551; found 366.9549.

1-(1-(4-Fluorophenylthio)propan-2-ylthio)-4-fluorobenzene,
Entry 9; Obtained as pale yellow liquid (elution with light petroleum).
IR (Nujol) 1589.2, 1488.9, 1230.5, 1157.2, 1091.6, 1006.0, 980.0, 825.5, 620.0, 513.0 cm^{-1}; ^1H NMR ($CDCl_3$, 300 MHz): δ 1.37 (d, *J* = 6.6 Hz, 3H, CH_3), 2.73-2.78 (m, 1H, CH), 3.06-3.15 (m, 2H, CH_2), 6.90-7.00 (m, 4H, ArH), 7.18-7.29 (m, 4H, ArH), ^{13}C NMR ($CDCl_3$, 75 MHz): δ 19.2, 41.7, 41.8, 43.1, 115.8, 115.82, 116.09, 116.1, 128.7, 128.8, 130.5, 130.54, 132.5, 132.6, 135.3, 135.4, 160.2, 160.8, 163.4, 164.1; HRMS (ESI) calcd for $C_{15}H_{14}F_2KS_2$ 335.0142; found 335.0144.

1-(3-(4-Fluorophenylthio)propylthio)-4-fluorobenzene,
Entry 10; Obtained as pale yellow liquid (elution with light petroleum).
IR (Nujol) 1589.2, 1488.9, 1226.6, 1157.2, 1091.6, 1014.5, 825.5, 628.8, 513.0 cm^{-1}; ^1H NMR ($CDCl_3$, 300 MHz): δ 1.85 (quintet, *J* = 6.9 Hz, 2H, CH_2), 2.96 (t, *J* = 6.9 Hz, 4H, $2CH_2$), 6.94-7.00 (m, 4H, ArH), 7.28-7.33 (m, 4H, ArH); ^{13}C NMR ($CDCl_3$, 75 MHz): δ 28.3, 33.7, 115.8, 116.1, 130.6, 130.7, 132.5, 132.6, 160.2, 163.4; HRMS (ESI) calcd for $C_{15}H_{14}F_2KS_2$ 335.0142; found 335.0143.

2-(1-(2,6-Ddimethylphenylthio)propan-2-ylthio)-1,3-dimethylbenzene,
Entry 11; Obtained as colourless liquid (elution with light petroleum).
IR (Nujol) 1600.8, 1558.4, 1485.1, 1176.5, 1064.6, 1041.5, 1006.8, 879.5, 810.0, 690.0, 550.0, 451.3 cm^{-1}; 1H NMR ($CDCl_3$, 300 MHz): δ 1.45 (d, *J* = 6.6 Hz, 3H, CH_3), 2.20 (s, 3H, $ArCH_3$), 2.23 (s, 3H, $ArCH_3$), 2.29 (s, 3H,$ArCH_3$), 2.35 (s, 3H, $ArCH_3$), 2.71-2.80 (m, 1H, CH), 3.21-3.26 (m, 2H, CH_2), 6.88-7.09 (m, 6H, ArH); ^{13}C NMR ($CDCl_3$, 75 MHz): δ 19.4, 19.9, 20.3, 20.77, 20.8, 39.9, 41.6, 126.9, 128.2, 129.5, 130.1, 130.3, 133.1, 133.3, 134.4, 135.0, 135.9, 137.0.

2-(3-(2,6-Dimethylphenylthio)propylthio)-1,3-dimethylbenzene,
Entry 12; Obtained as colourless liquid (elution with light petroleum).
IR (Nujol) 1600.8, 1060.8, 806.2 cm^{-1}; ^{1}H NMR ($CDCl_3$, 300 MHz): δ 1.99 (quintet, *J* = 6.9 Hz, 2H, CH_2), 2.28-2.32 (s, 12H, $4CH_3$), 3.03 (t, *J* = 6.9 Hz, 4H, $2CH_2$), 6.89 (d, *J* = 6.9 Hz, 2H, ArH), 7.03-7.07 (m, 4H, ArH). ^{13}C NMR ($CDCl_3$, 75 MHz): δ 19.9, 21.0, 28.2, 31.9, 126.6, 128.7, 130.0, 134.5, 135.0, 135.9; HRMS (ESI) calcd for $C_{19}H_{24}K_2S_2$ 394.0594; found 394.0597.

1-(2-(Pentylthio)propylthio)pentane,
Entry 13; Obtained as pale yellow liquid (elution with light petroleum).
IR (Nujol) 1168.8 cm^{-1}; ^{1}H NMR ($CDCl_3$, 300 MHz): δ 0.88-0.92 (m, 6H, $2CH_3$), 1.29-1.39 (m, 8H, $4CH_2$), 1.35 (d, *J* = 6.9 Hz, 3H, CH_3), 1.54-1.61 (m, 4H, $2CH_2$), 2.50-2.58 (m, 5H, CH & 2 S-CH_2), 2.82-2.88 (m, 2H, S-C$\underline{H}_2$-CH); ^{13}C NMR ($CDCl_3$, 75 MHz): δ 13.9, 20.2, 22.2, 29.3, 29.4, 30.6, 30.9, 31.1, 33.9, 39.6, 39.8; HRMS (ESI) calcd for $C_{13}H_{28}KS_2$ 287.1269; found 287.1271.

1-(3-(Pentylthio)propylthio)pentane,
Entry 14; Obtained as colourless liquid (elution with light petroleum).
IR (Nujol) 1253.6 cm^{-1}; ^{1}H NMR ($CDCl_3$, 300 MHz): δ 0.90 (t, *J* = 6.9 Hz, 6H, $2CH_3$), 1.29-1.41 (m, 8H, 4CH2), 1.56-1.61 (m, 4H, $2CH_2$), 1.86 (quintet, *J* = 6.9 Hz, 2H, CH_2), 2.51 (t, *J* = 7.2 Hz, 4H, $2CH_2$), 2.59-2.62 (m, 4H, $2CH_2$); ^{13}C NMR ($CDCl_3$, 75 MHz): δ 14.0, 22.3, 29.3, 29.4, 31.0, 31.1, 32.1; HRMS (ESI) calcd for $C_{13}H_{28}KS_2$ 287.1269; found 287.1270.

(1-(Cyclohexylthio)propan-2-ylthio)cyclohexane [3],
Entry 15; Obtained as pale yellow liquid (elution with light petroleum).
IR (Nujol) 1265.2, 995.2, 887.2, 817.8, 721.3 cm^{-1}; ^{1}H NMR ($CDCl_3$, 300 MHz): δ 1.29-1.32 (m, 10H, CyH), 1.34 (d, *J* = 6.6 Hz, 3H, CH_3), 1.62 (s, 2H, CyH), 1.76 (s, 4H, CyH), 1.95 (s, 4H, CyH), 2.49-2.75 (m, 3H, 2CyH, CH), 2.84-2.97 (m, 2H, CH_2); ^{13}C NMR ($CDCl_3$, 75 MHz): δ 20.7, 25.6, 25.9, 25.94, 33.5, 33.6, 33.7, 33.9, 37.8, 38.2, 42.4, 44.2; HRMS (ESI) calcd for $C_{15}H_{28}KS_2$ 311.1269; found 311.1271.

(3-(Cyclohexylthio)propylthio)cyclohexane,

Entry 16; Obtained as colourless liquid (elution with light petroleum).

IR (Nujol) 1203.5, 999.1, 883.3, 817.8, 717.5 cm^{-1}; ^{1}H NMR ($CDCl_3$, 300 MHz): δ 1.24-1.41 (m, 11H, Cy-H), 1.60-1.99 (m, 12H, Cy-H & CH_2), 2.63 (t, J = 7.2 Hz, 4H, 2 S-CH_2), 2.97-2.99 (m, 2H, 2 S-CH); ^{13}C NMR ($CDCl_3$, 75 MHz): δ 25.7, 25.8, 25.9, 26.1, 29.0, 30.1, 33.66, 33.7, 37.8, 33.9, 43.4, 43.5, 44.7, 49.3; HRMS (ESI) calcd for $C_{15}H_{28}KS_2$ 311.1269; found 311.1270.

1-(1-(2-Methoxyphenylthio)propan-2-ylthio)-2-methoxybenzene,

Entry 17; Obtained as pale yellow liquid (elution with 3% ethyl acetate - light petroleum).

IR (Nujol) 1577.7, 1296.1, 1272.9, 1242.1, 1180.4, 1130.2, 1072.3, 1026.1, 833.2, 794.6, 748.3, 721.3, 675.0, 525.0 cm^{-1}; ^{1}H NMR ($CDCl_3$, 300 MHz): δ 1.42 (d, J = 6.9 Hz, 3H, CH_3), 2.64-2.72 (m, 1H, CH), 3.24-3.37 (m, 2H, CH_2), 3.70 (s, 3H, OCH_3), 3.71 (s, 3H, OCH_3), 6.74-6.84 (m, 4H, ArH), 7.07-7.23 (m, 4H, ArH); ^{13}C NMR ($CDCl_3$, 75 MHz): δ 18.8, 38.7, 39.8, 55.2, 110.2, 110.4, 120.49, 120.5, 121.8, 123.1, 127.3, 128.4, 130.3, 133.2, 157.4, 158.4; HRMS (ESI) calcd for $C_{17}H_{20}O_2NaS_2$ 343.0802; found 343.0801.

2-(1-(Naphthalene-6-ylthio)propan-2-ylthio)naphthalene,

Entry 18; Obtained as white crystalline solid (elution with 2% ethyl acetate - light petroleum), mp. 91-93 ^{o}C.

IR (KBr) 817.8, 740.6, 478.3 cm^{-1}; ^{1}H NMR ($CDCl_3$, 300 MHz): δ 1.47 (d, J = 6.9 Hz, 3H, CH_3), 2.82-2.90 (m, 1H, CH), 3.37-3.44 (m, 2H, CH_2), 7.23 (d, J = 8.4 Hz, 1H, ArH), 7.33-7.44 (m, 6H, ArH), 7.48-7.55 (m, 3H, ArH), 7.61 (d, J = 8.4 Hz, 1H, ArH), 7.67-7.74 (m, 3H, ArH); ^{13}C NMR ($CDCl_3$, 75 MHz): δ 19.2, 40.5, 42.4, 125.7, 126.1, 126.4, 126.43, 127.0, 127.3, 127.5, 127.57, 127.6, 128.38, 128.4, 129.7, 130.9, 131.5, 131.7, 132.2, 132.9, 133.5, 133.55, HRMS (ESI) calcd for $C_{23}H_{20}KS_2$ 399.0643; found 399.0645.

1-(3-(Phenylthio)propylthio)benzene and its 2-Deuterated product,

Entry 19; Obtained as colourless liquid mixture (elution with light petroleum).

^{1}H NMR ($CDCl_3$, 300 MHz): δ 1.90-1.99 (m, C<u>H</u>D & C<u>H</u>$_2$), 3.03-3.07 (m, 2CH_2, each from the mixture), 7.14-7.33 (m, 10 ArH, each from the mixture), ^{13}C NMR ($CDCl_3$, 75 MHz): δ 27.9 (t, J = 20 Hz, <u>C</u>HD), 28.3, 32.3, 32.4, 126.1, 128.9, 129.4, 136.0.

References:

1. Nagai, T.; Kumadaki, I.; Miki, T.; Kobayashi, Y.; Tomizawa, G. *Chem. Pharm. Bull.* **1986**, *34*, 1546–1552. http://dx.doi.org/10.1248/cpb.34.1546.
2. Abou, A.; Foubelo, F.; Miguel, Y. *Arkivoc*, **2007**, (iv), 196–205. http://www.arkat-usa.org/arkivoc-journal/browse-arkivoc/2007/4/
3. Anklam, E.; *Synthesis*, **1987**, 841. doi: 10.1055/2-1987-28097.
