# Supplementary material for: Silica: An efficient catalyst for one-pot regioselective synthesis of dithioethers
Source: Beilstein J Org Chem. 2014 Jan 7;10:26–33. doi: 10.3762/bjoc.10.5 (PMC3896229; doi:10.3762/bjoc.10.5)

Supporting Information

for

Silica: An efficient catalyst for one-pot regioselective synthesis of dithioethers

Samir Kundu[1], Babli Roy[1] and Basudeb Basu*[1]

Address: [1]Department of Chemistry, North Bengal University, Darjeeling 734013, India, Fax: +91 353 2699001

Email: Basudeb Basu* - basu_nbu@hotmail.com

*Corresponding author

^{1}H NMR and ^{13}C NMR spectra for compounds listed in Table 2, entries 1–19

Table 2; Entry 1

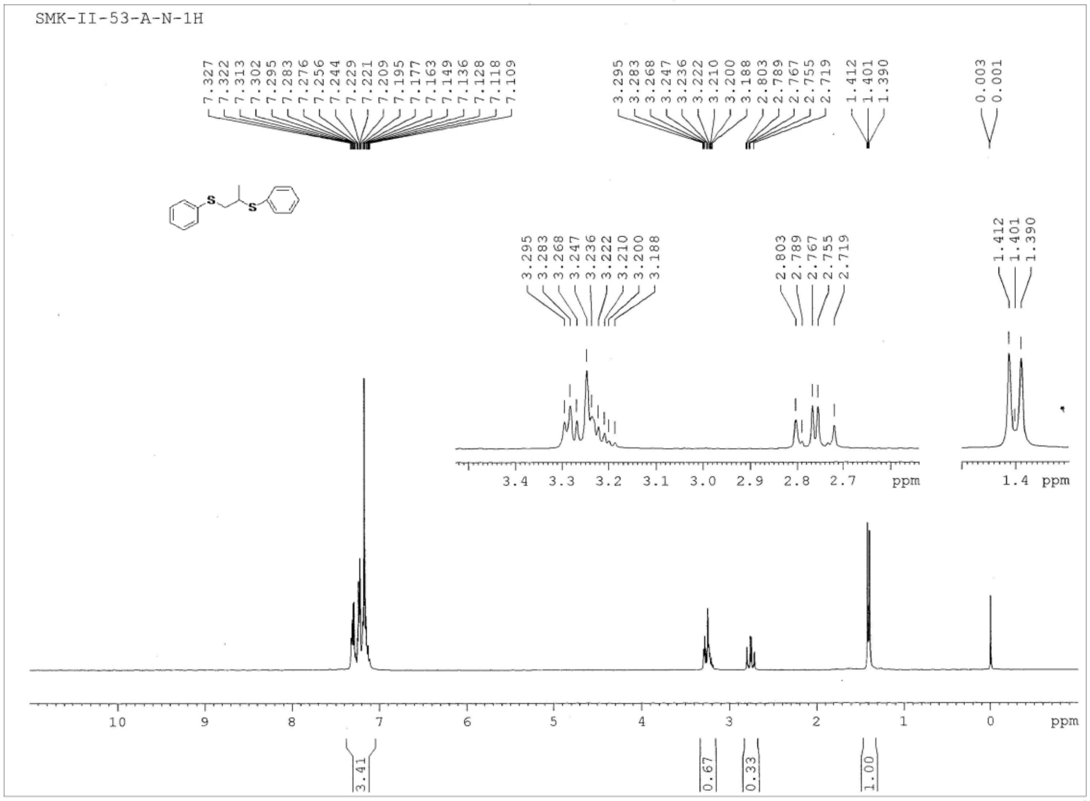

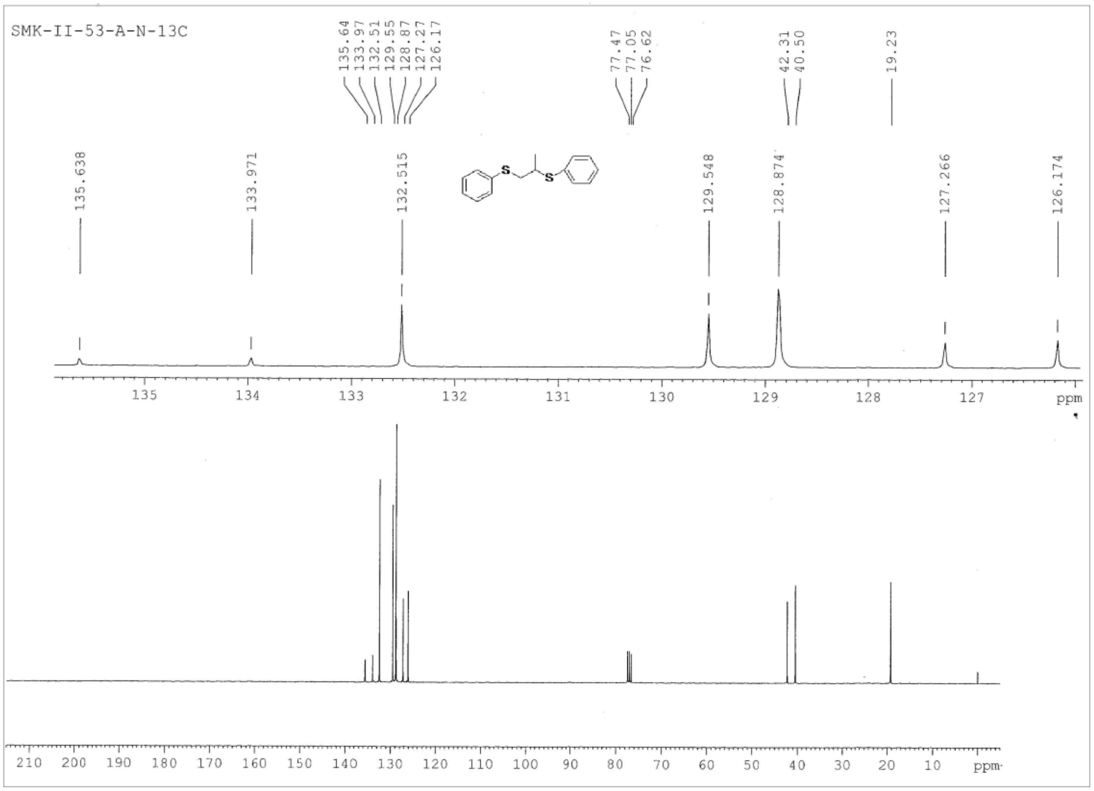

Table 2; Entry 2

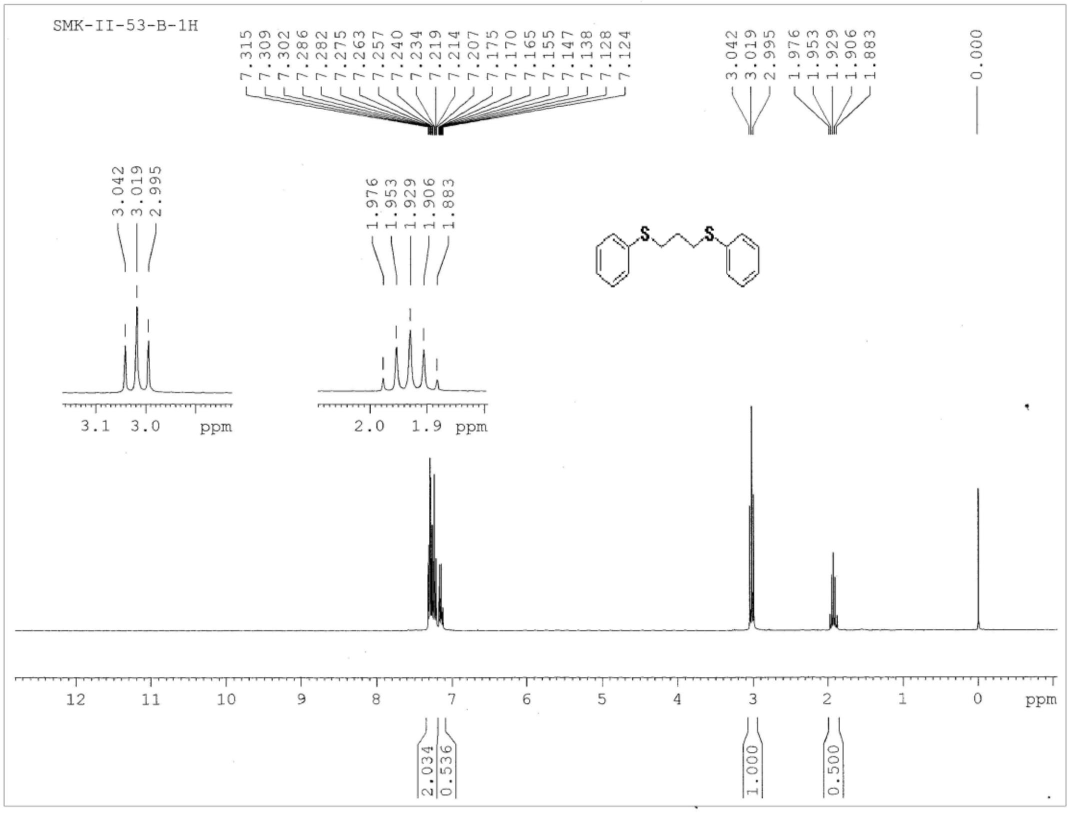

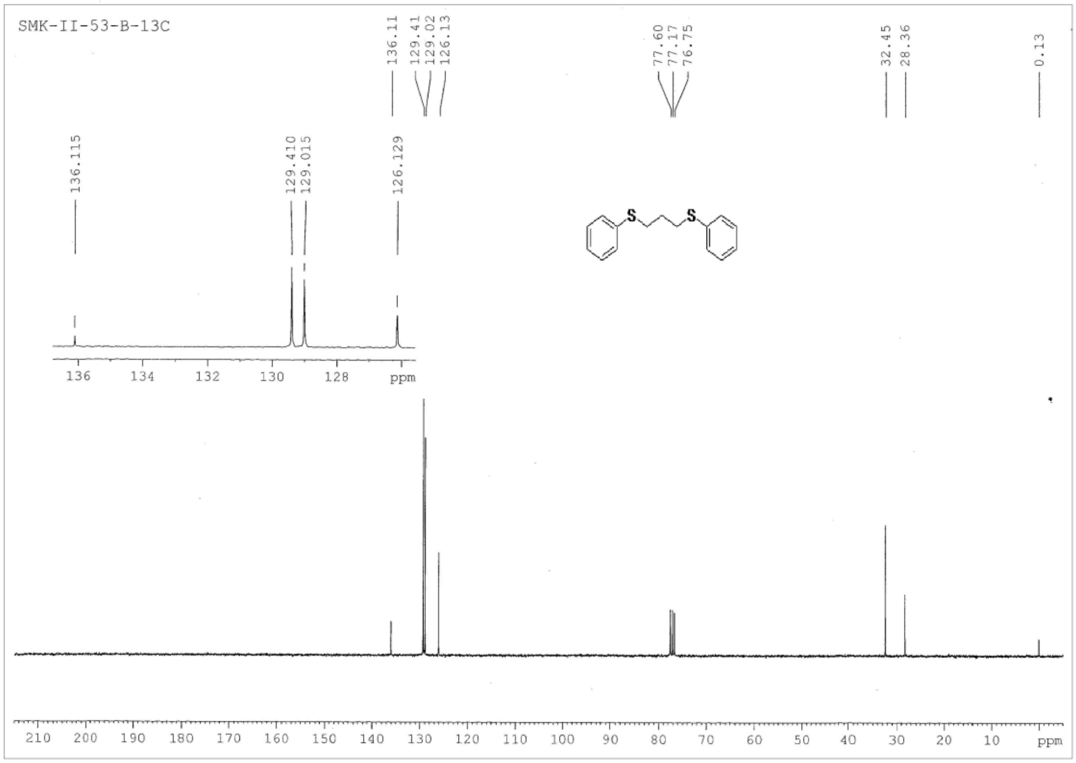

Table 2; Entry 3

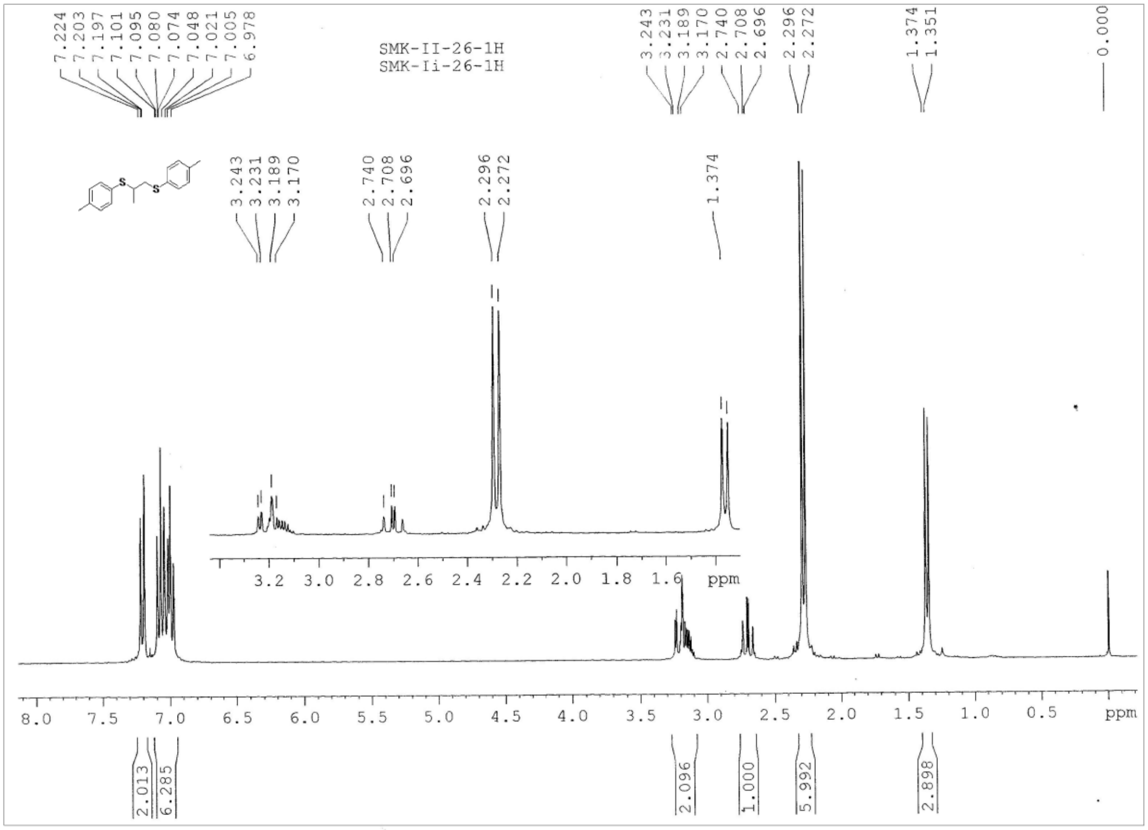

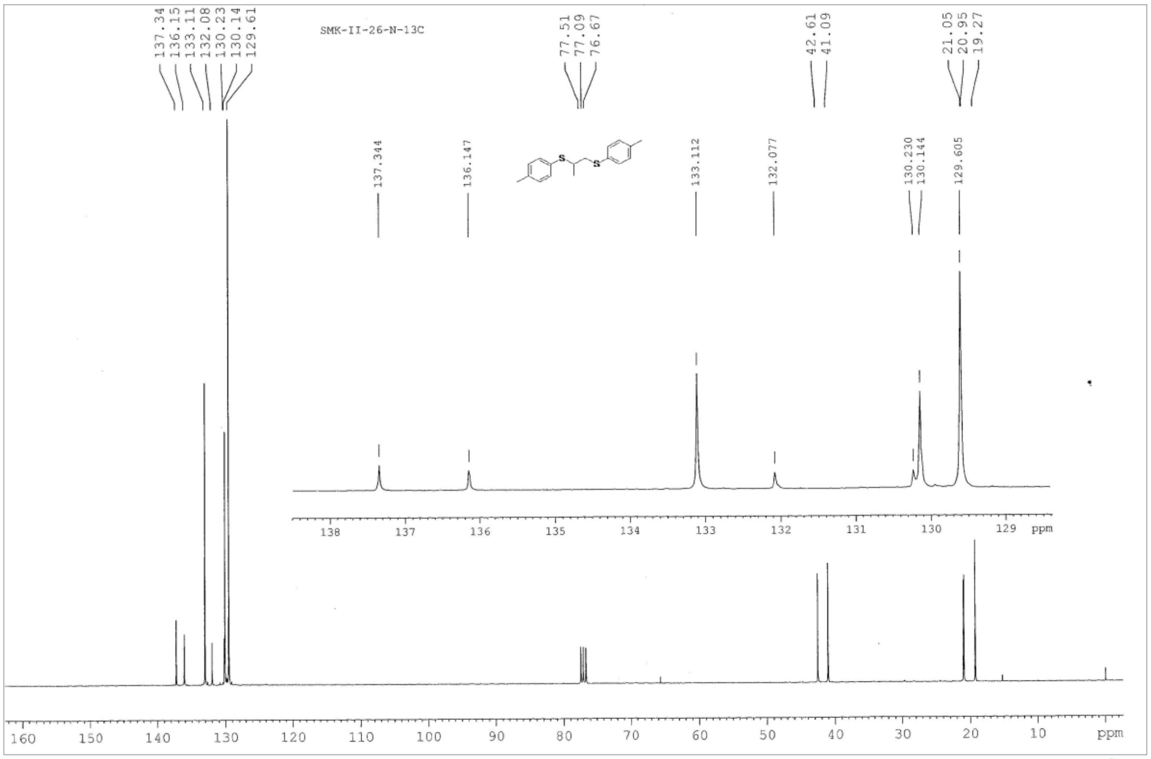

Table 2; Entry 4

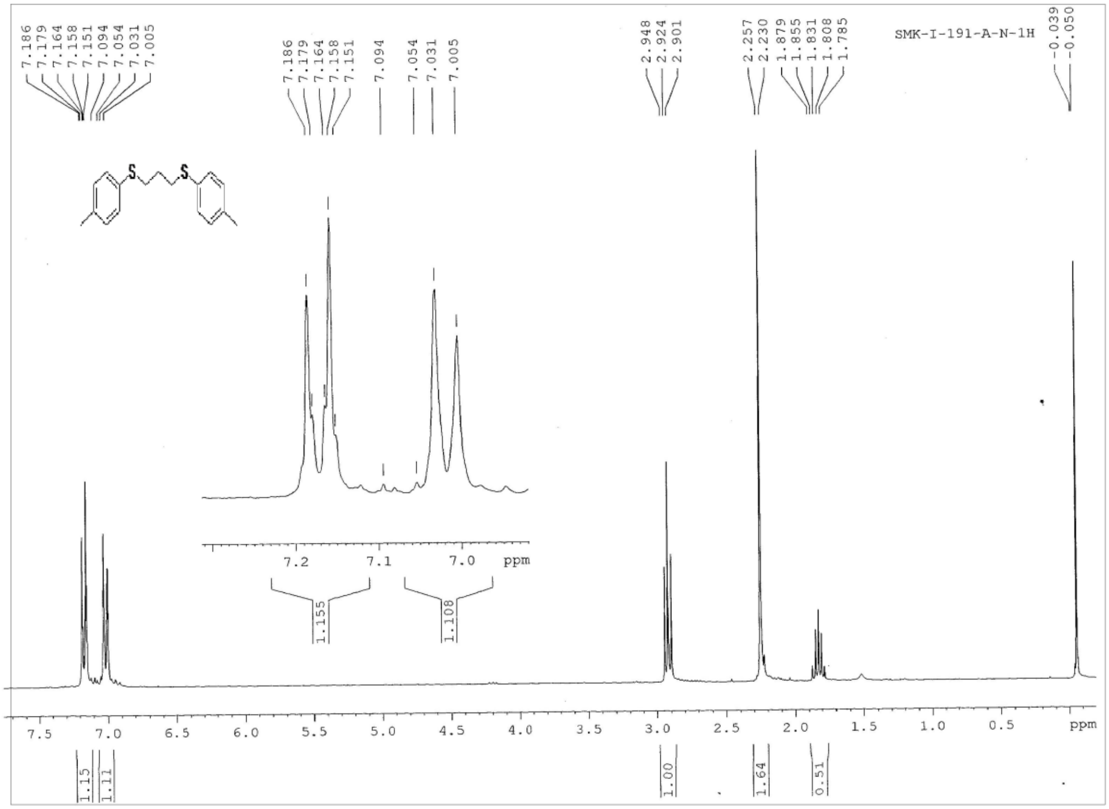

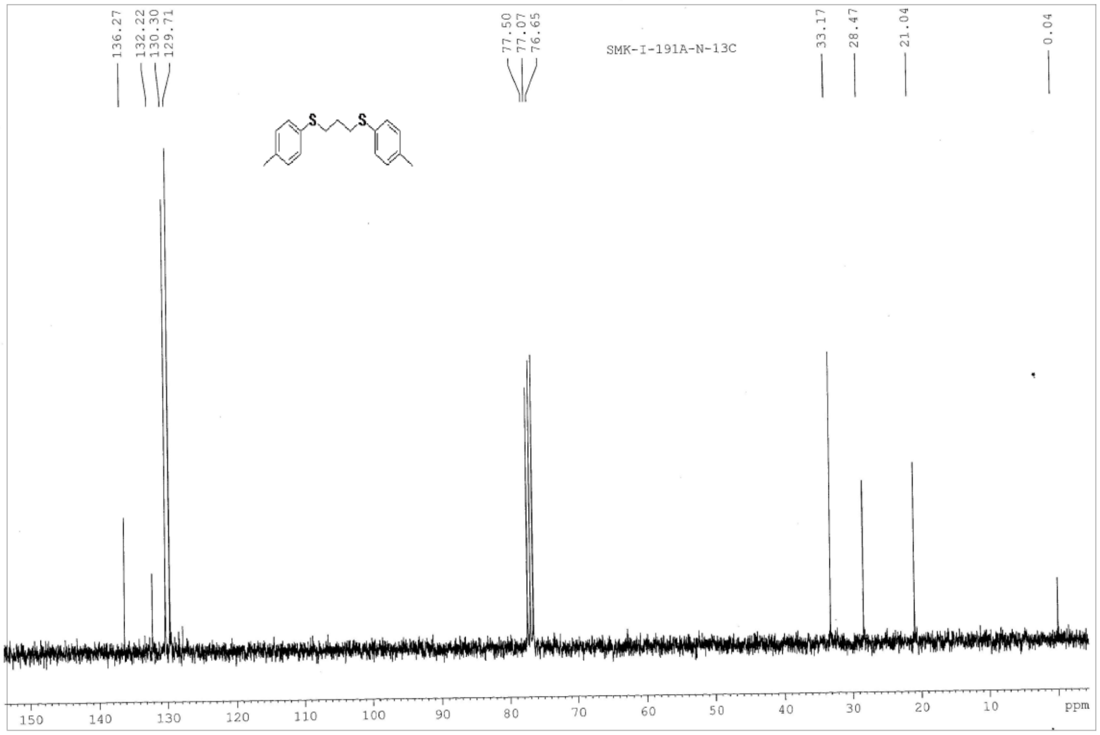

Table 2; Entry 5

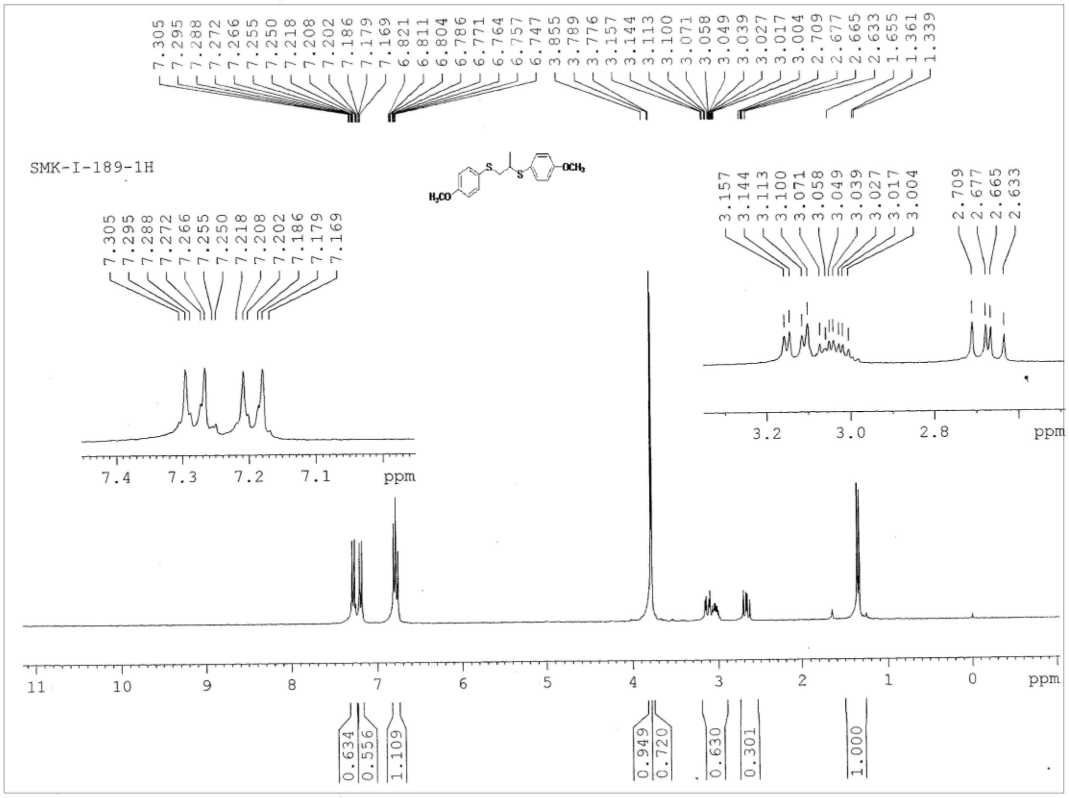

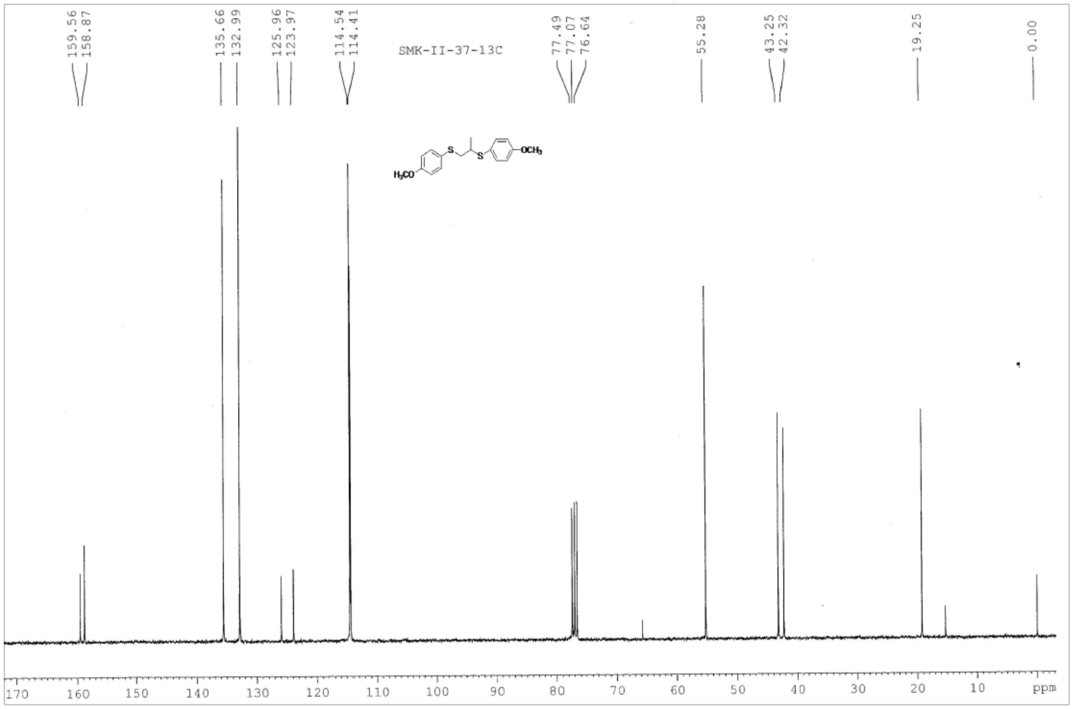

Table 2; Entry 6

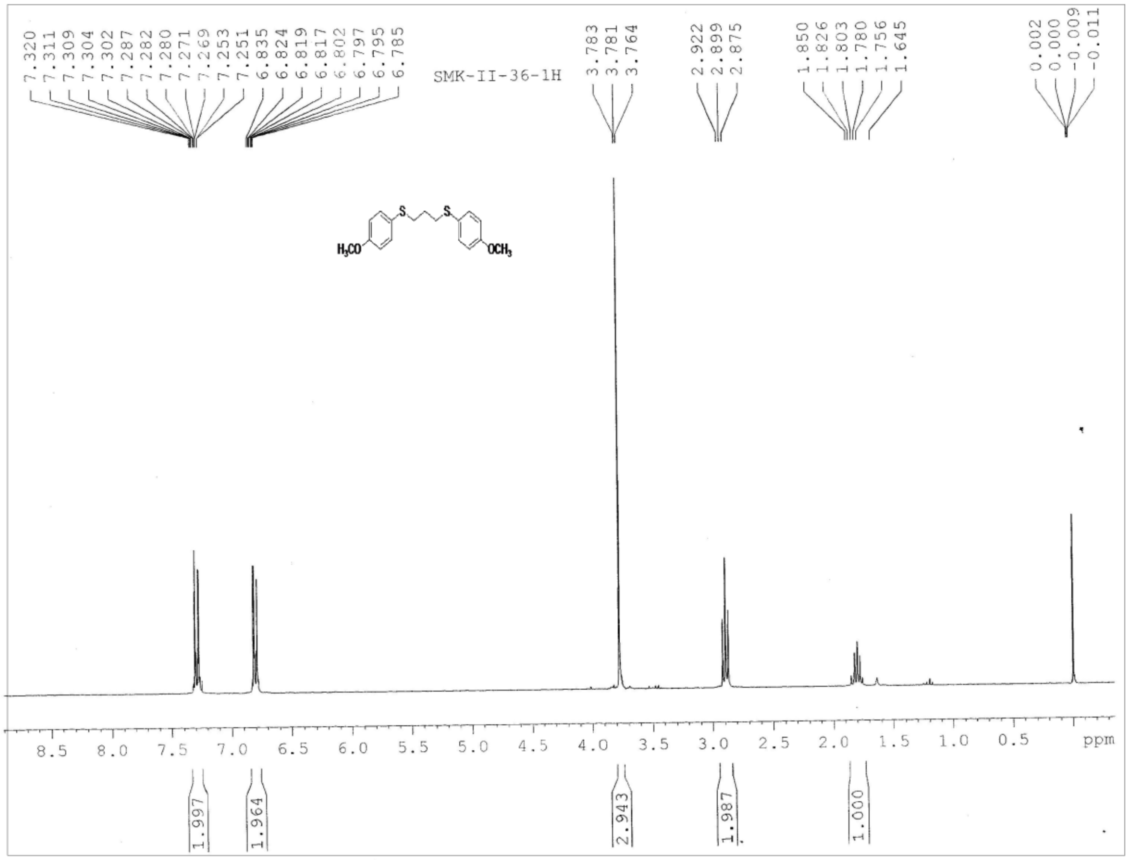

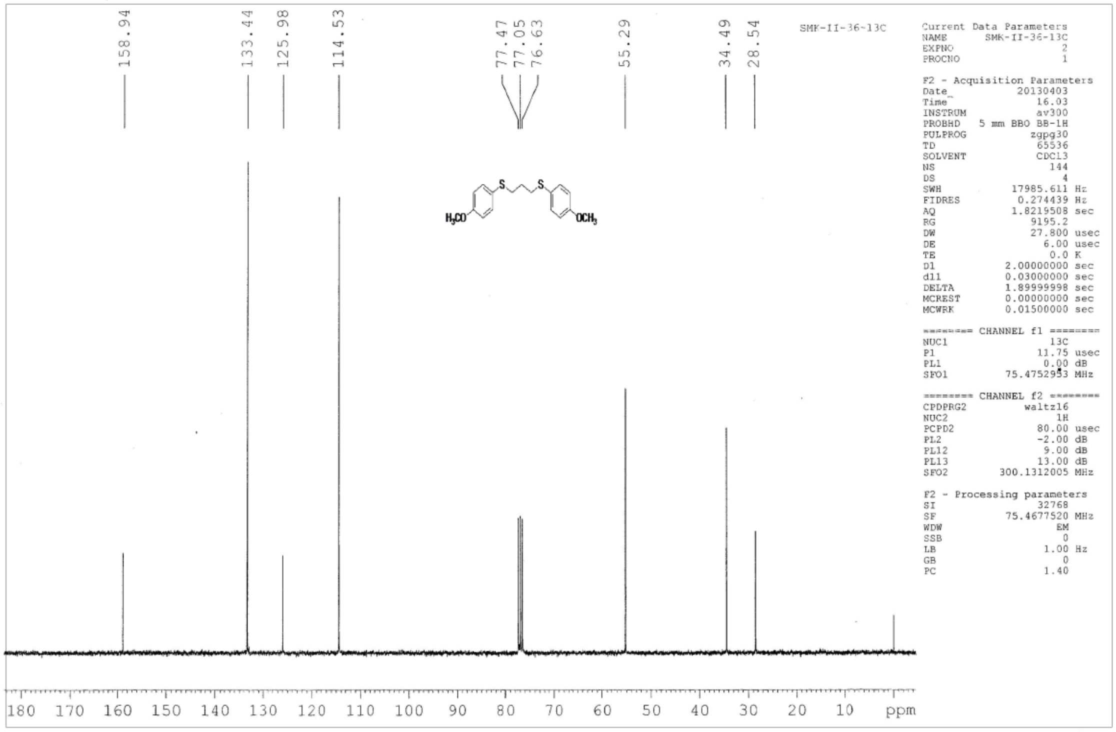

Table 2; Entry 7

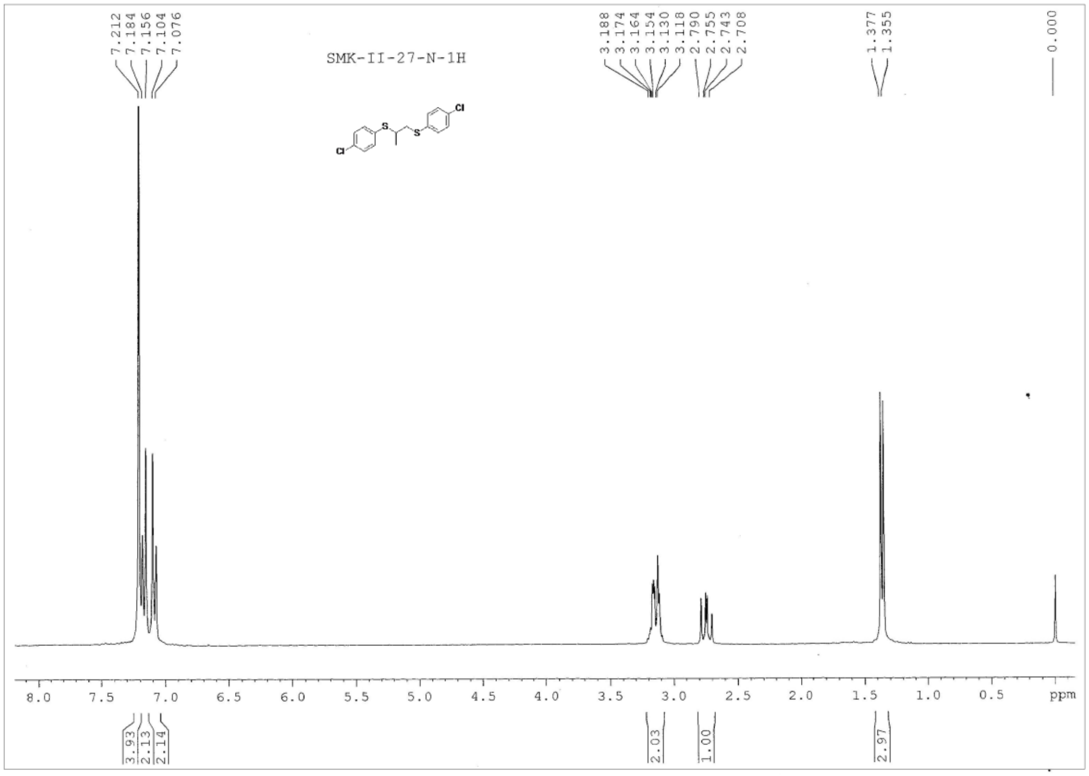

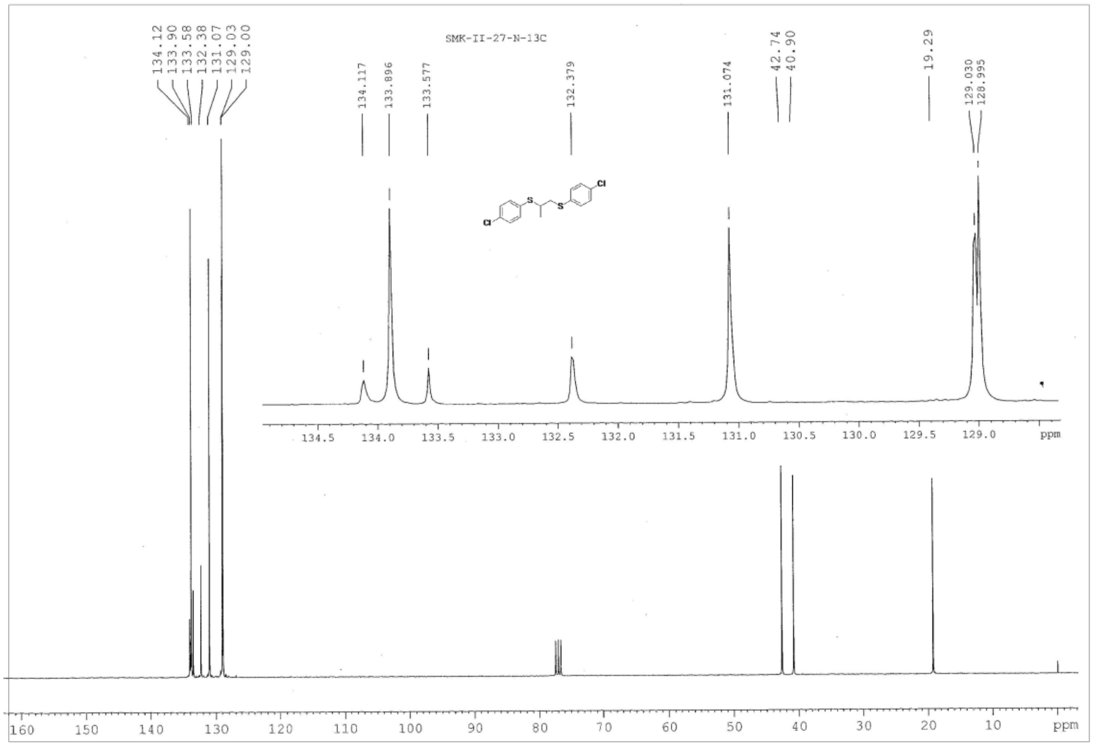

Table 2; Entry 8

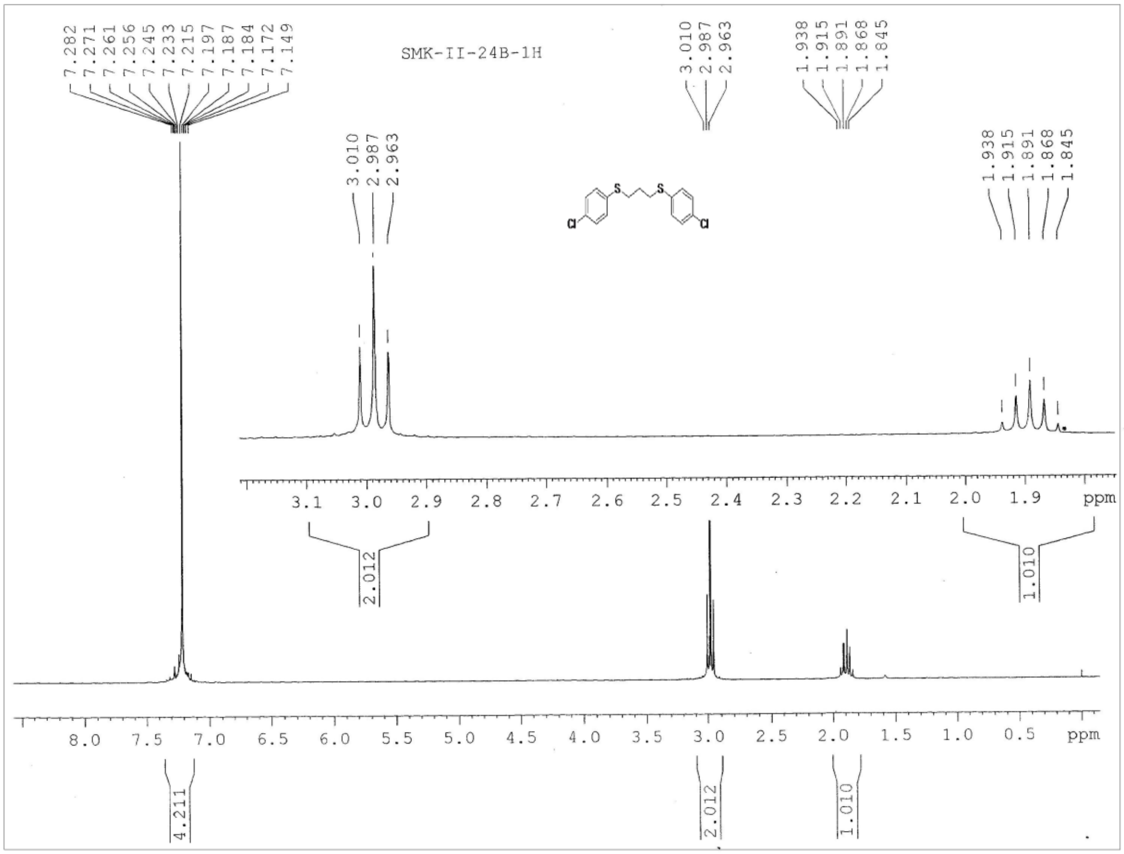

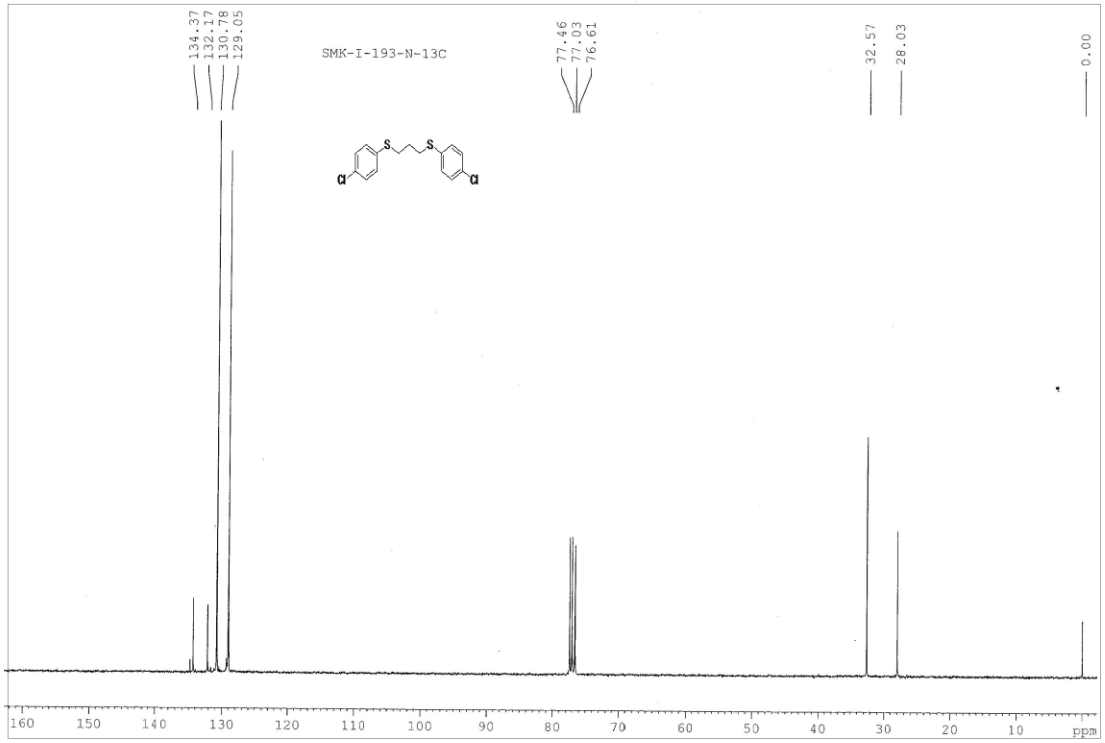

Table 2; Entry 9

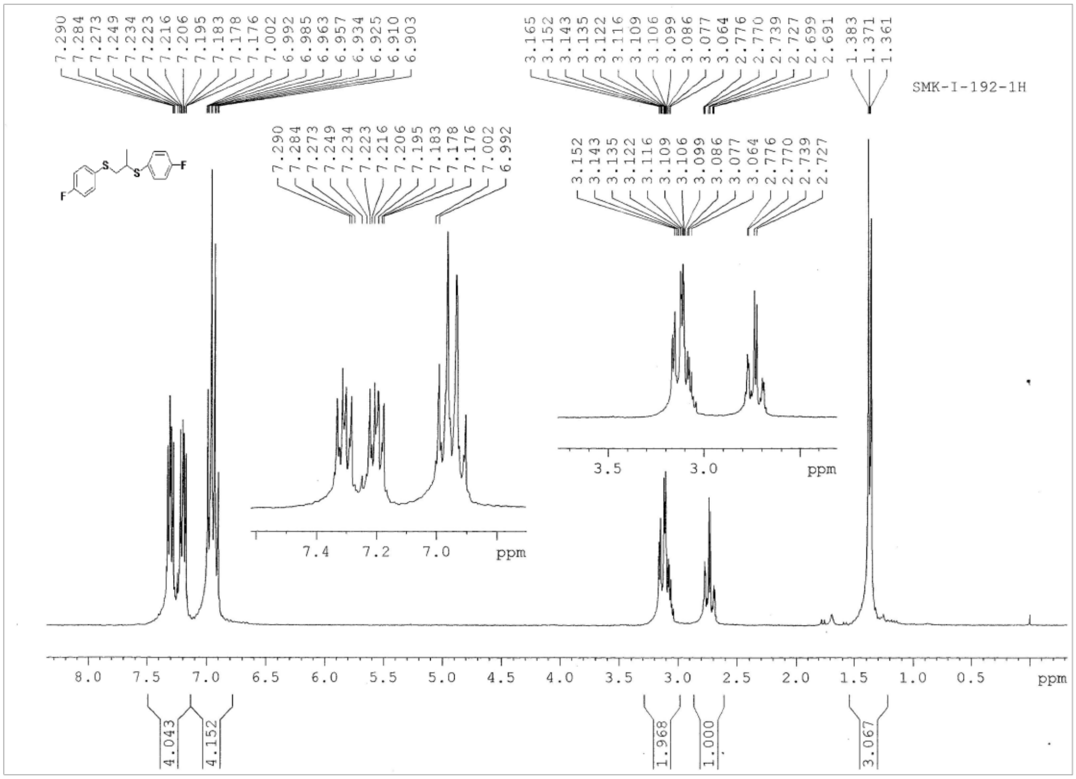

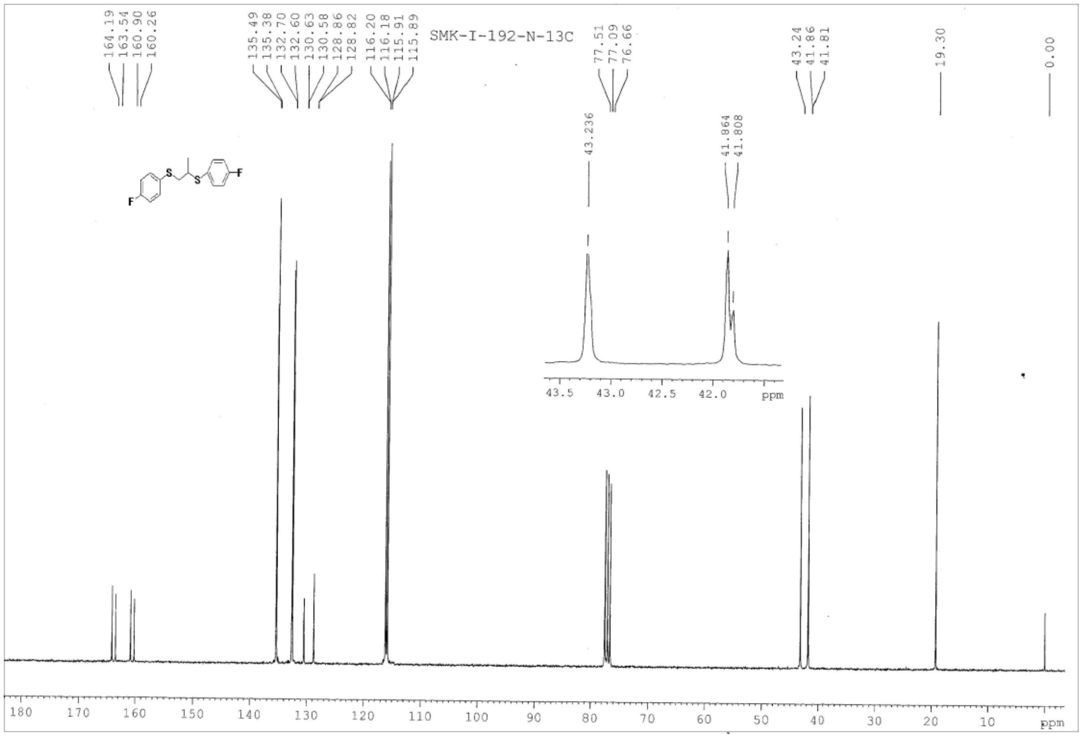

Table 2; Entry 10

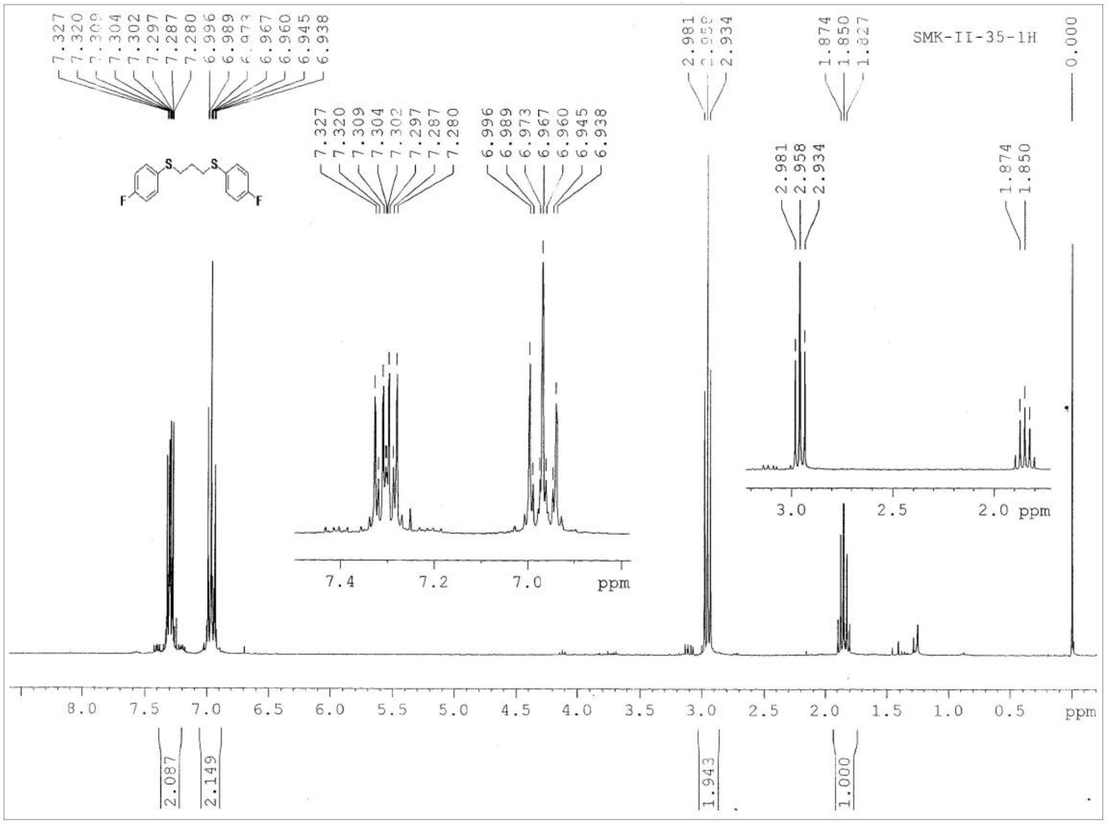

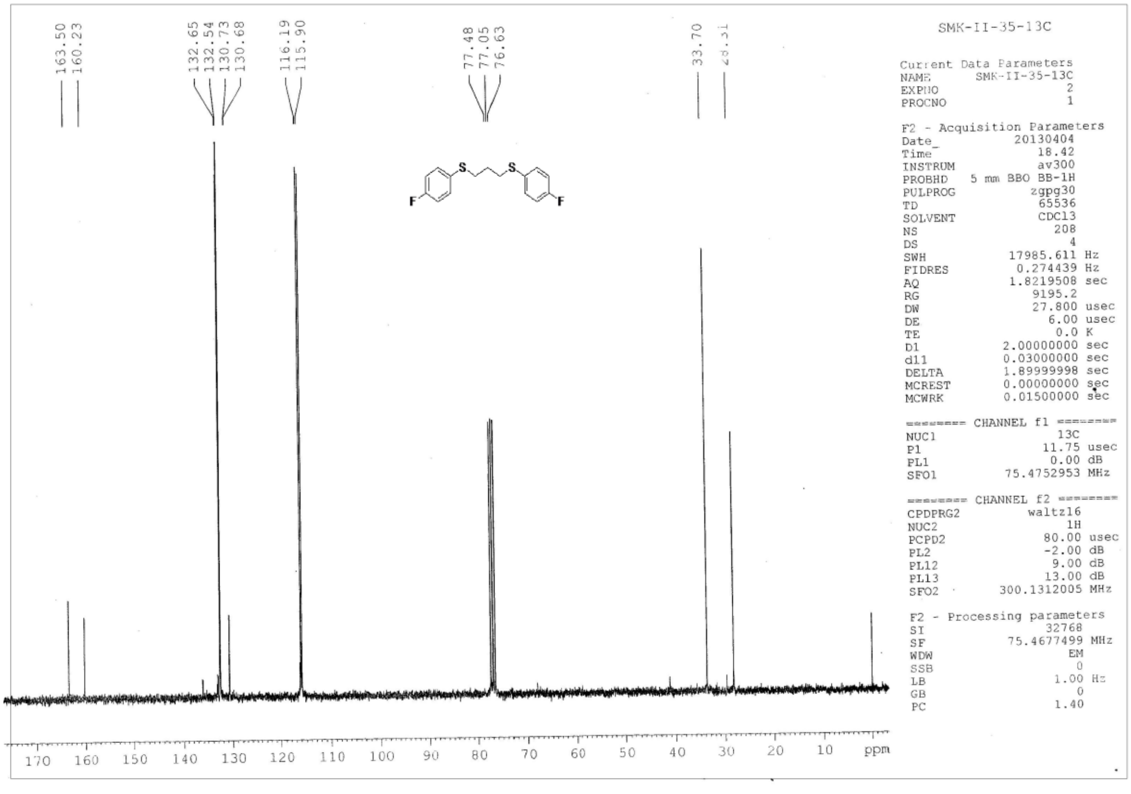

Table 2; Entry 11

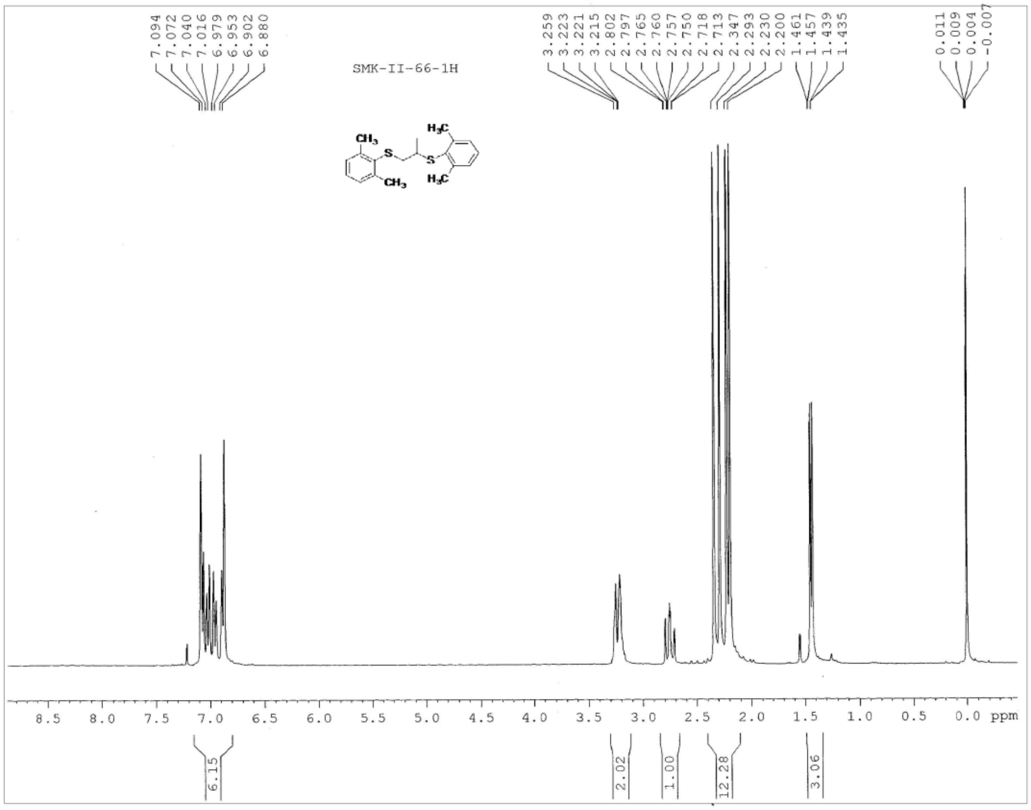

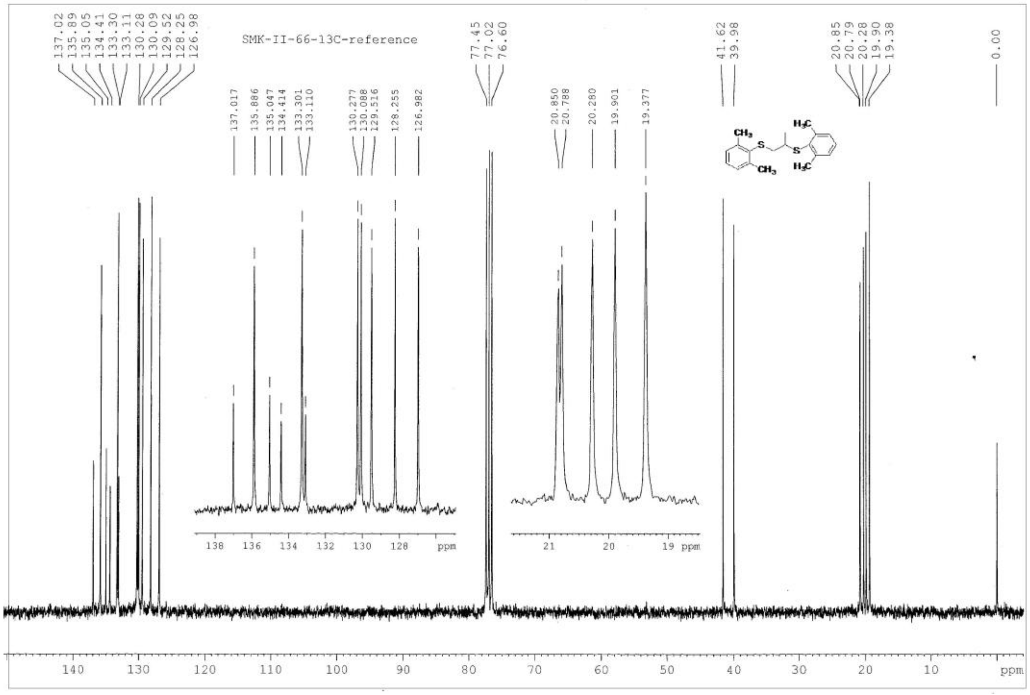

Table 2; Entry 12

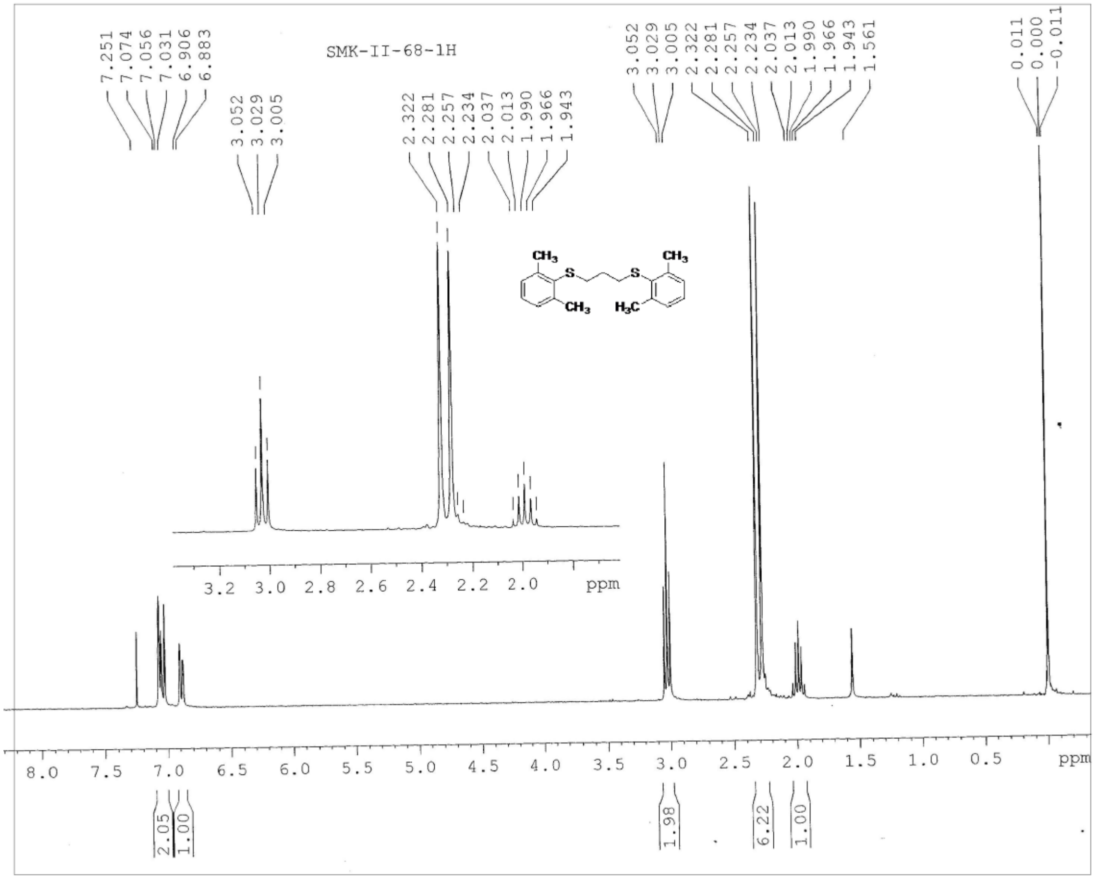

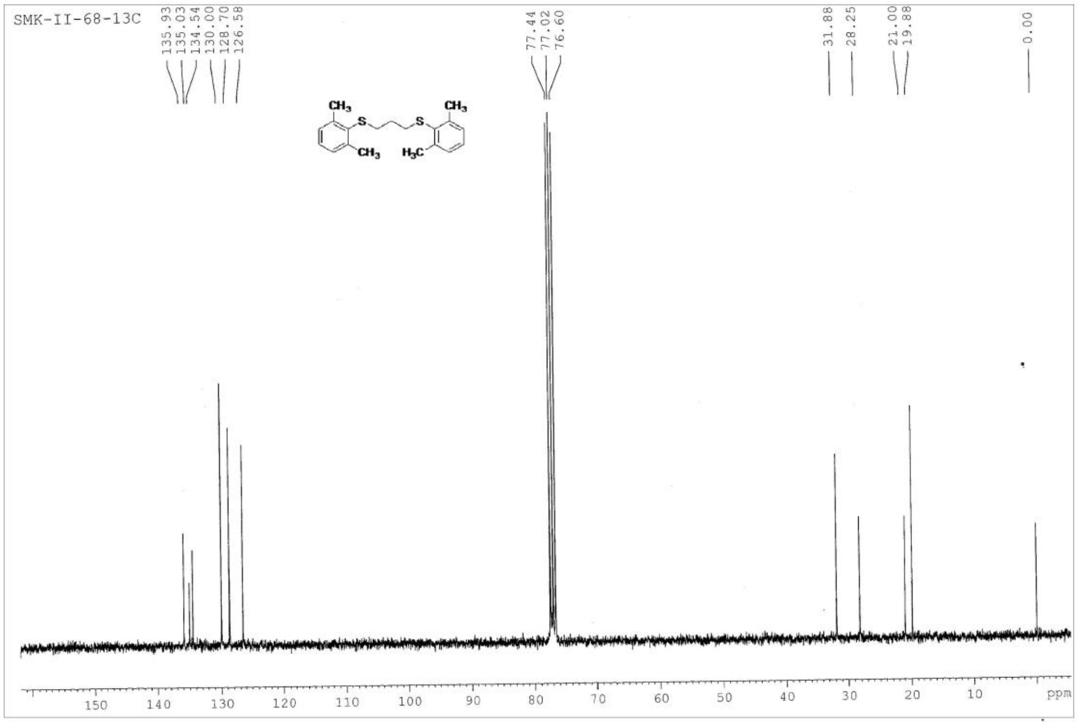

Table 2; Entry 13

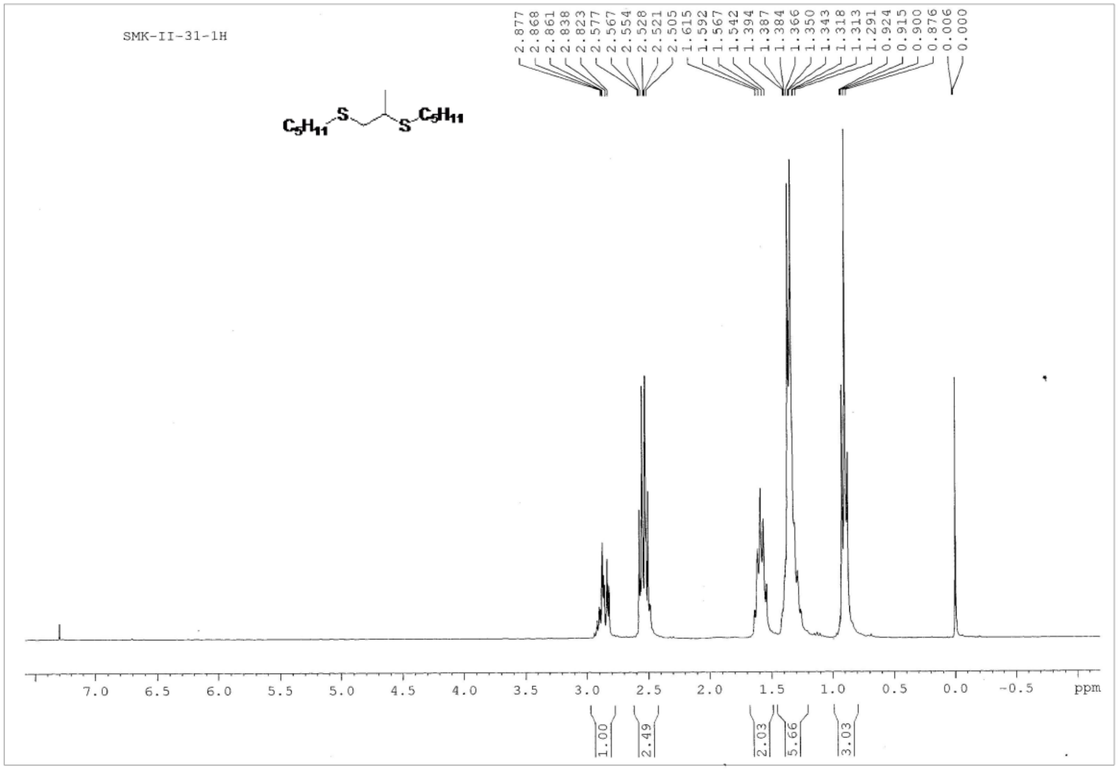

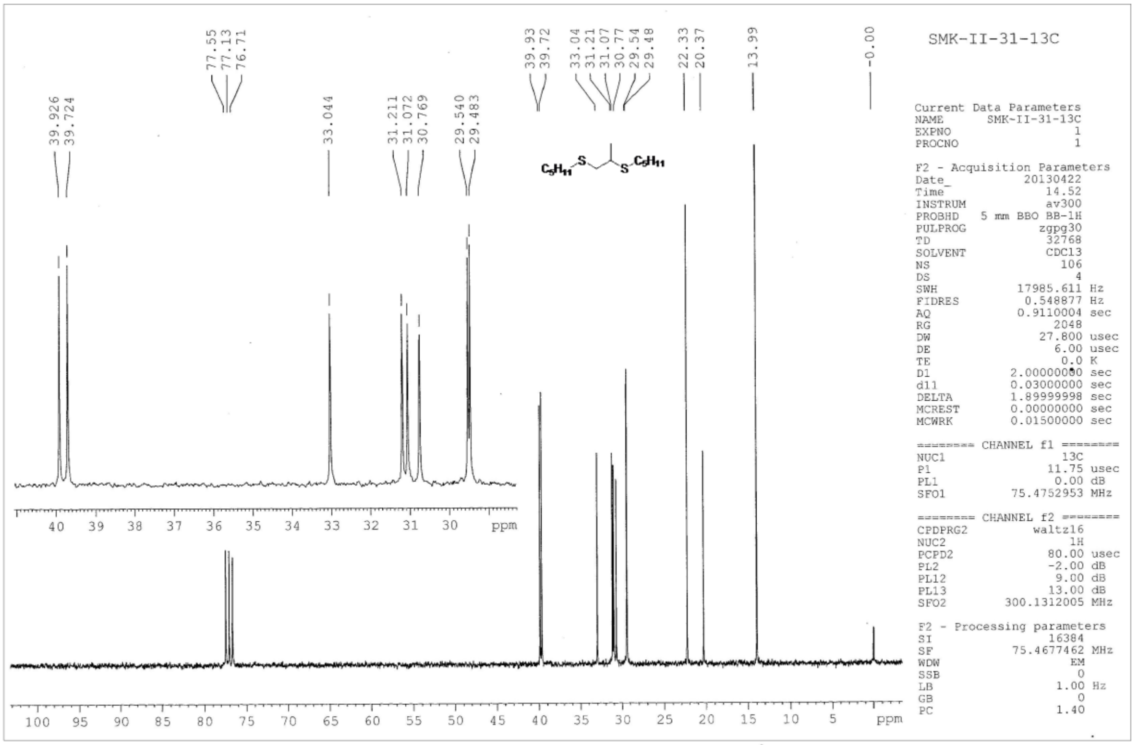

Table 2; Entry 14

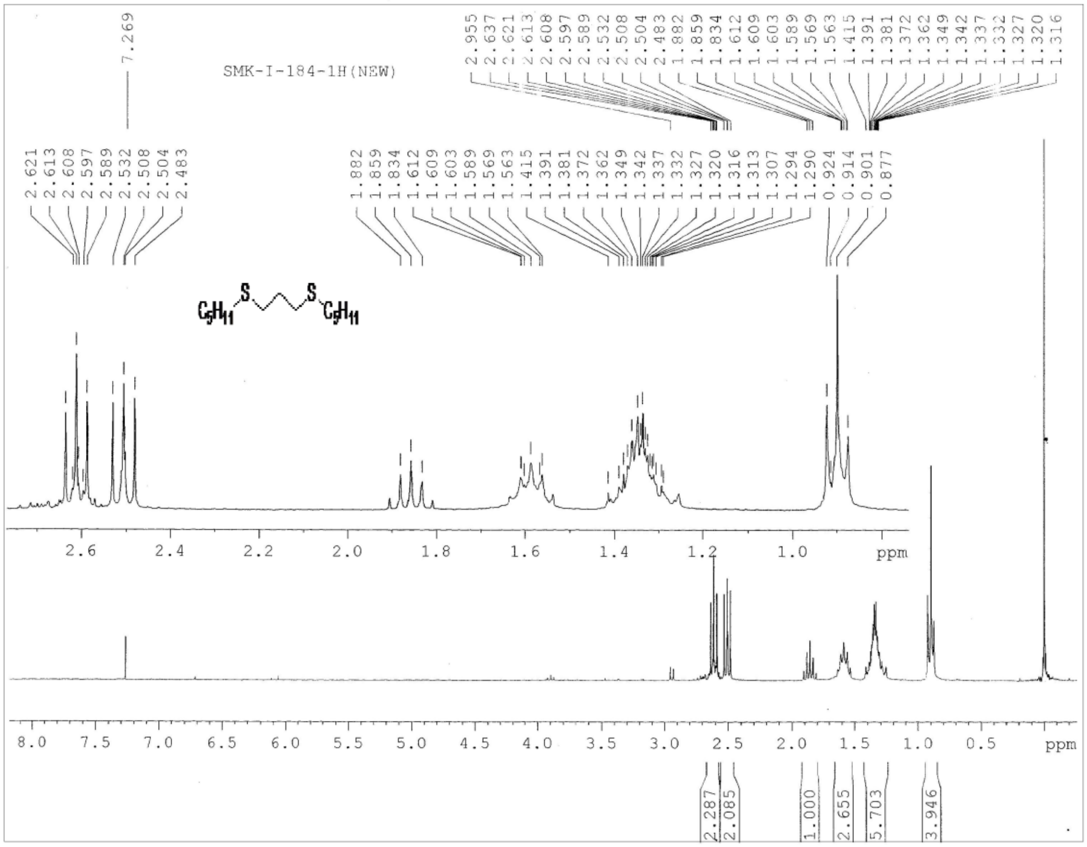

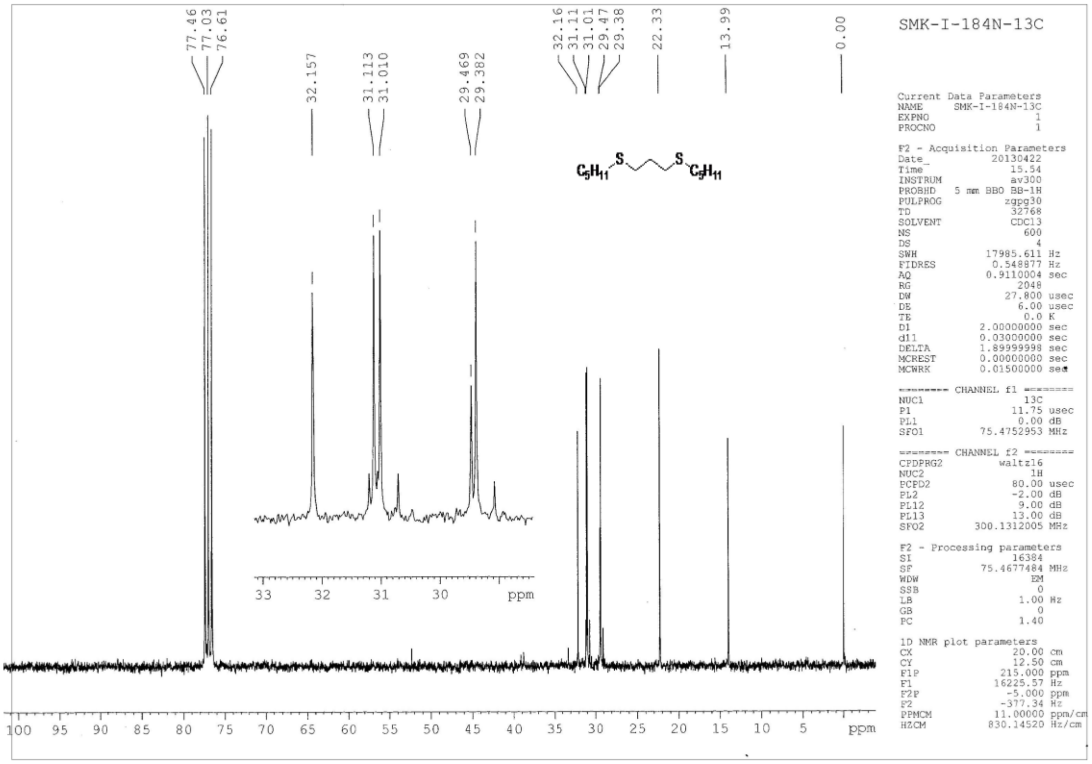

Table 2; Entry 15

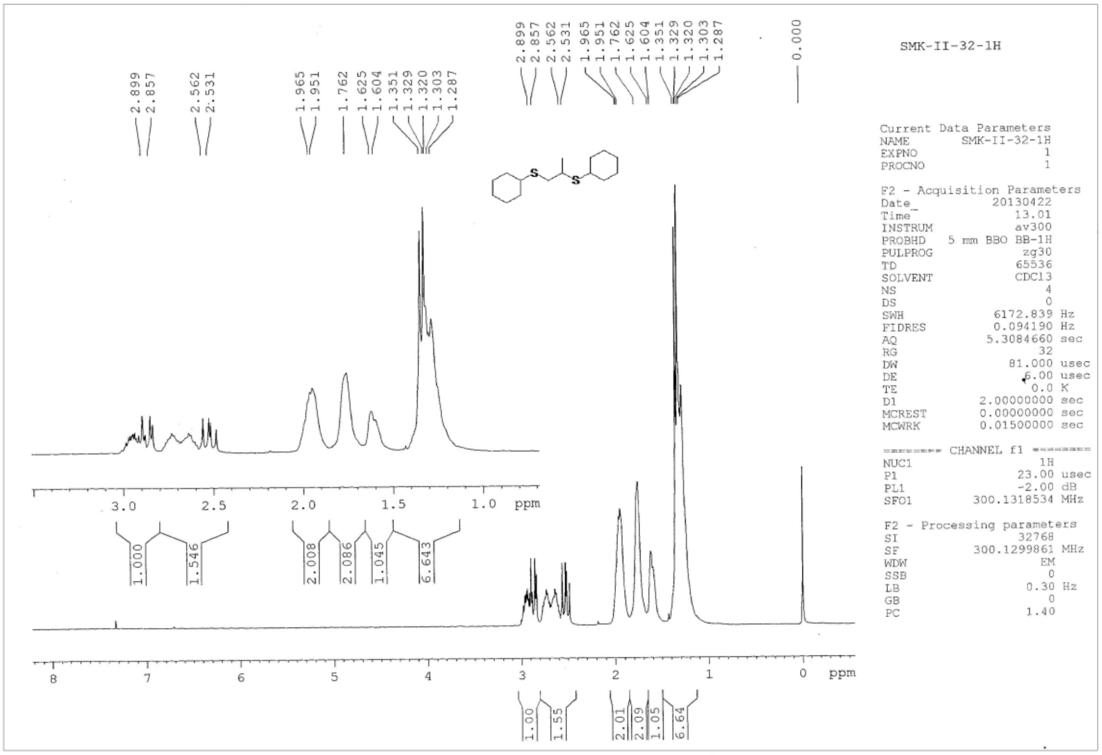

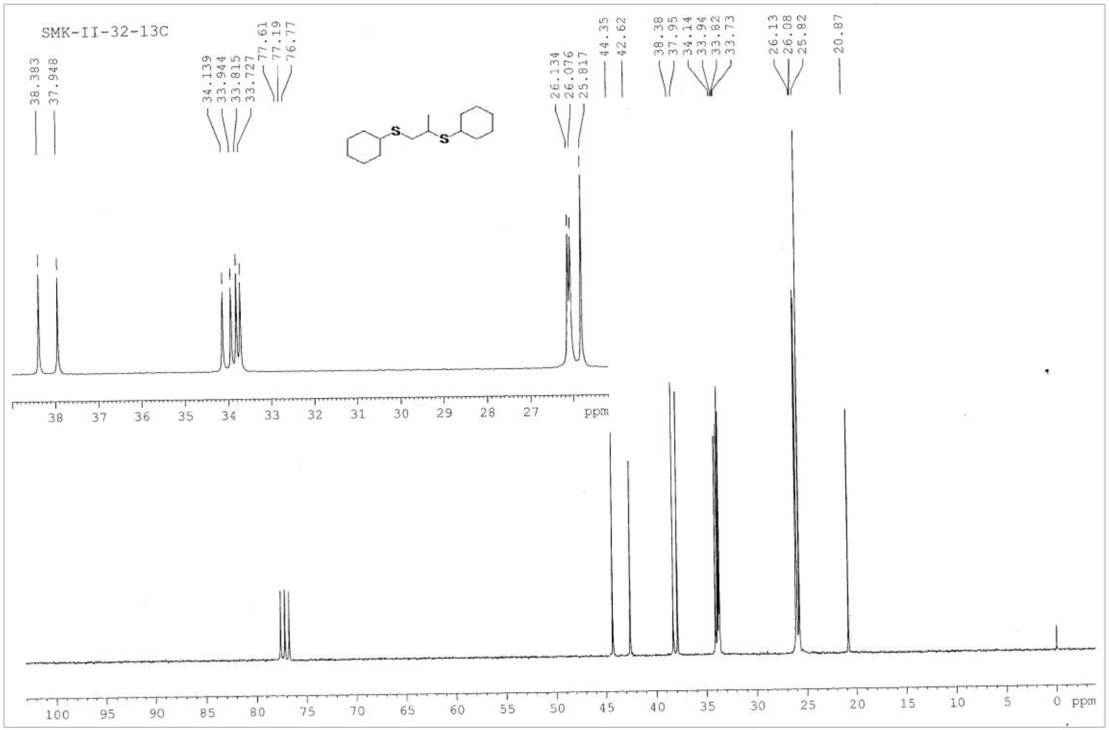

Table 2; Entry 16

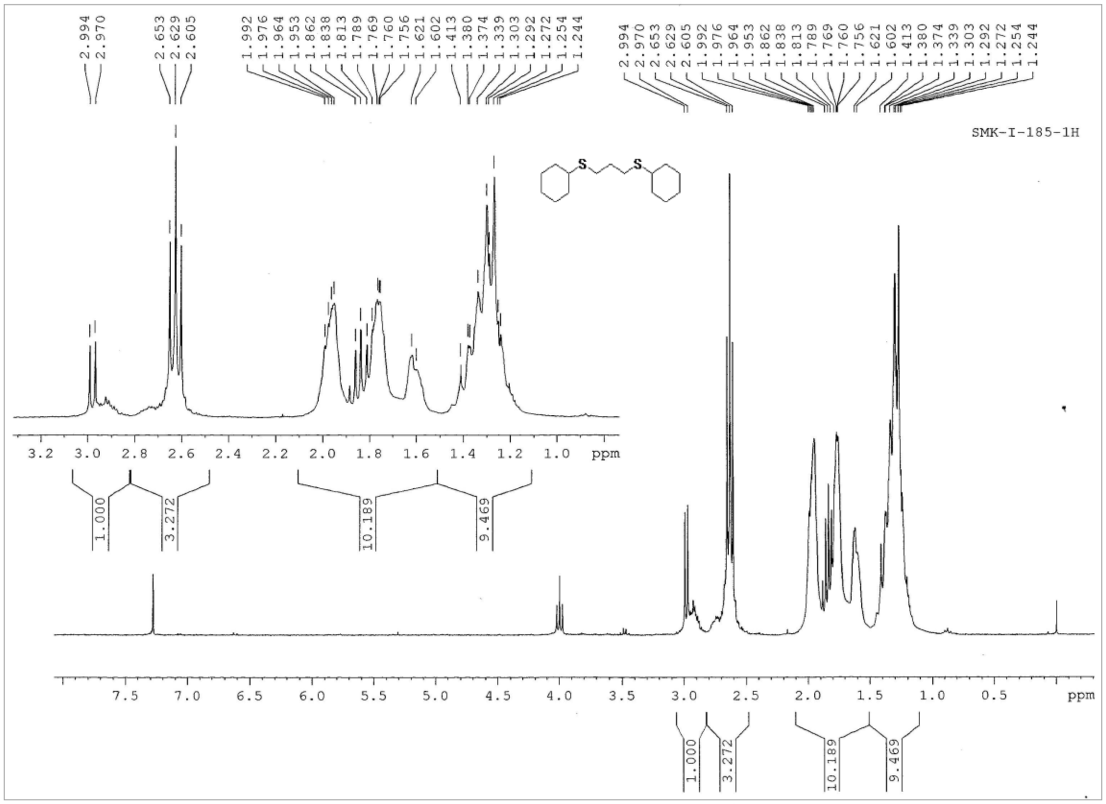

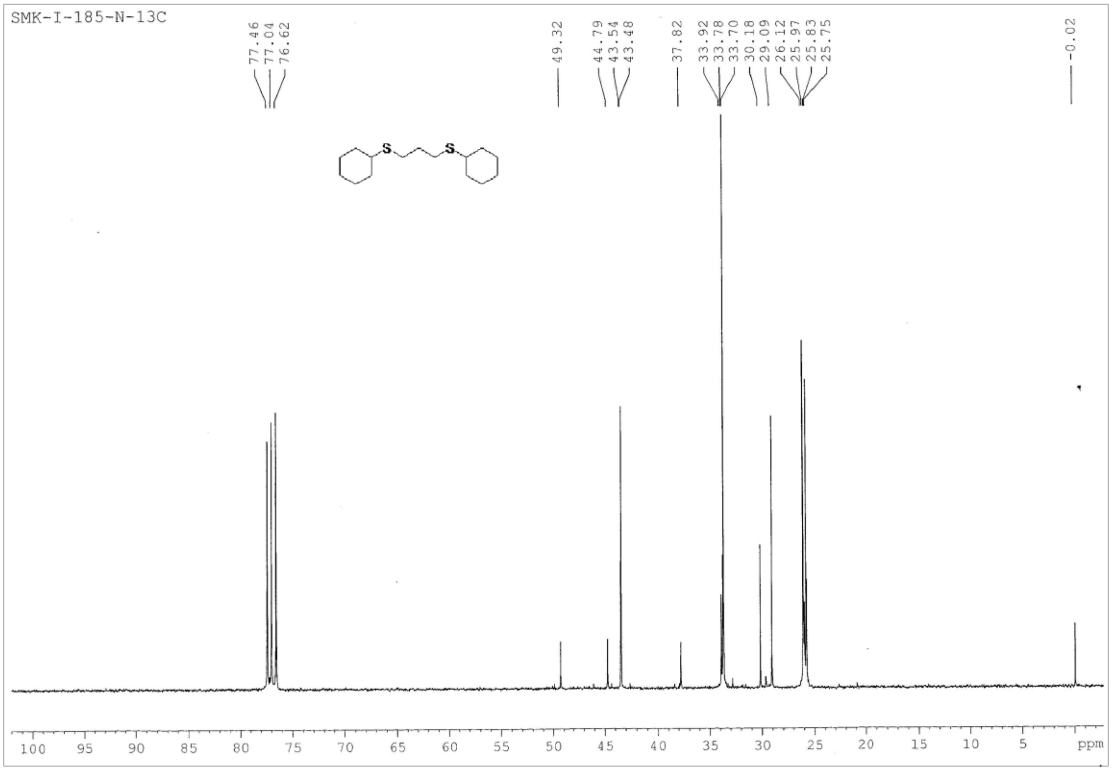

Table 2; Entry 17

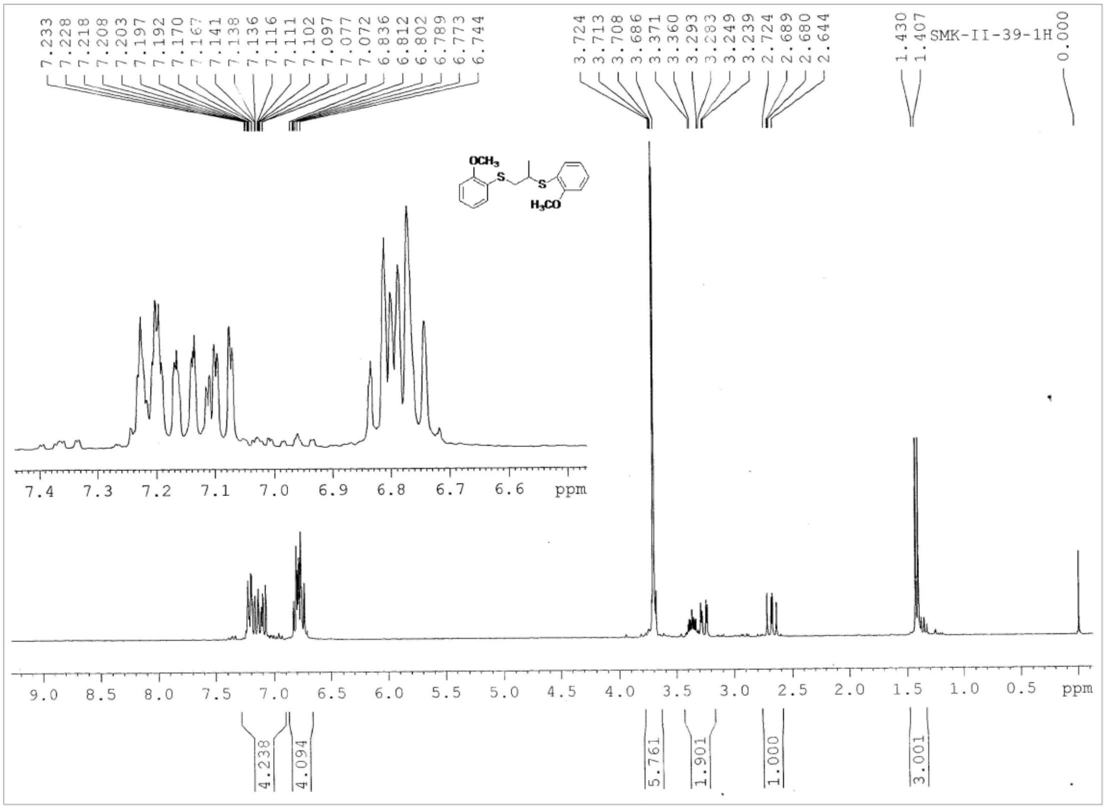

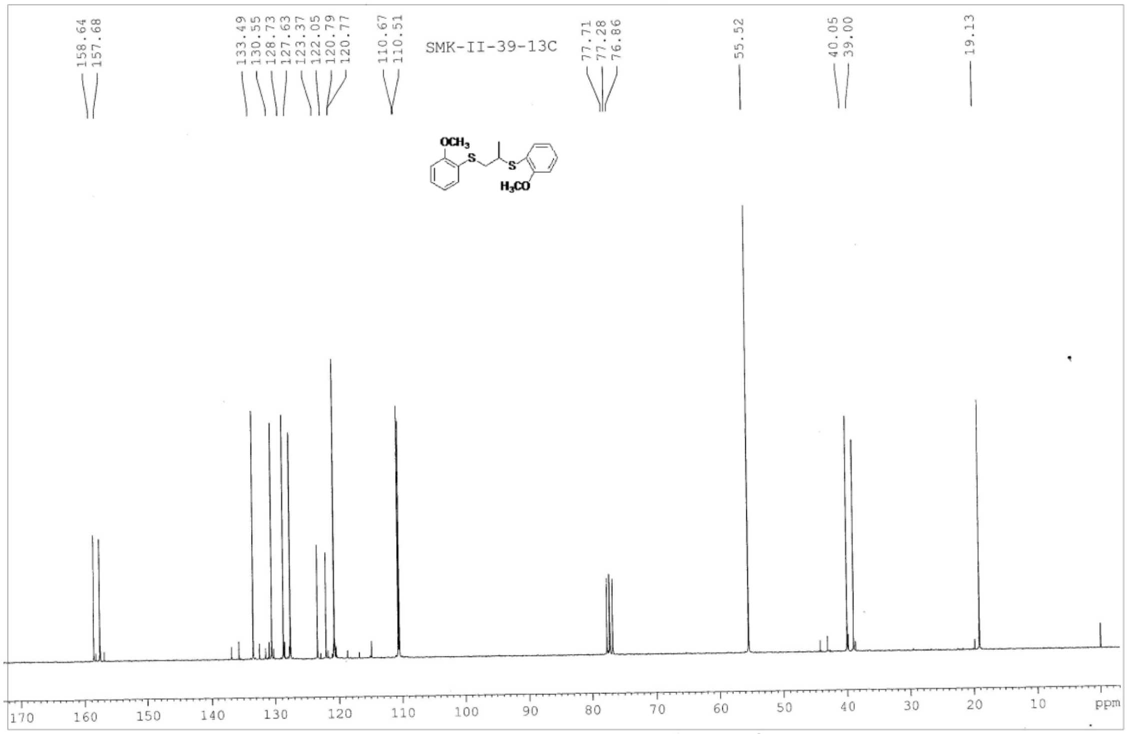

Table 2; Entry 18

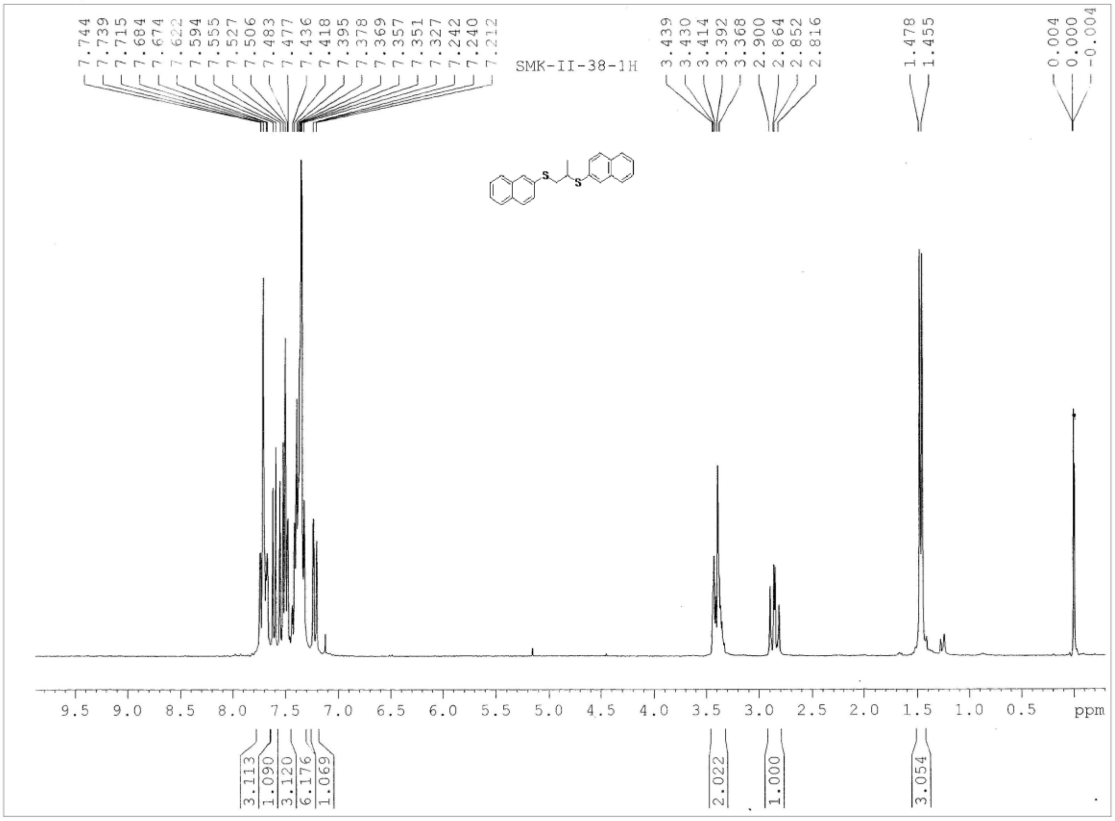

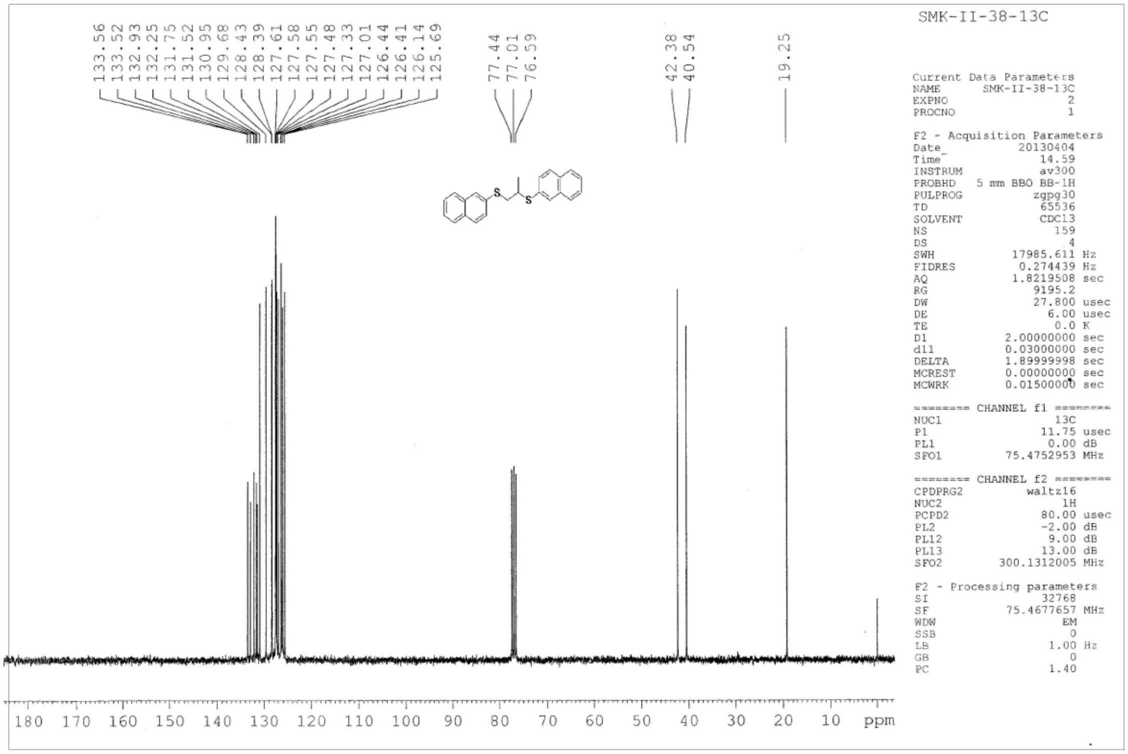

Table 2, Entry 19 (Mixture of 2-D-1,3-dithioether & the compound in Entry 2)

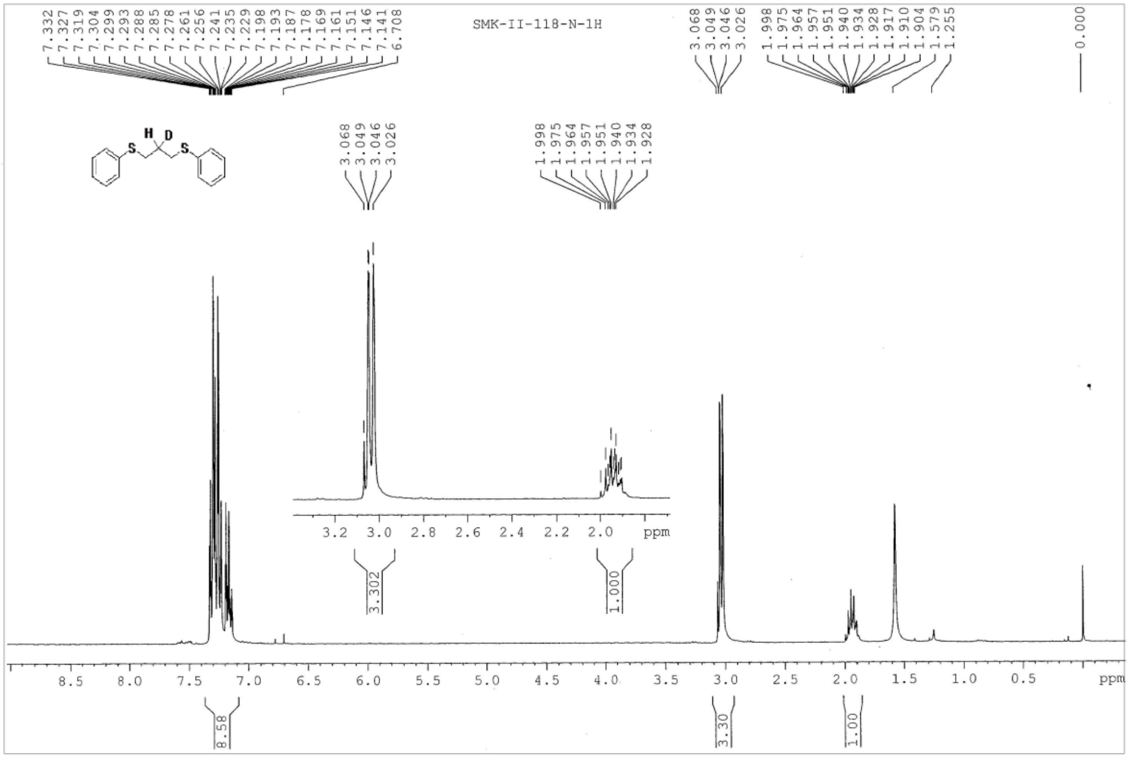

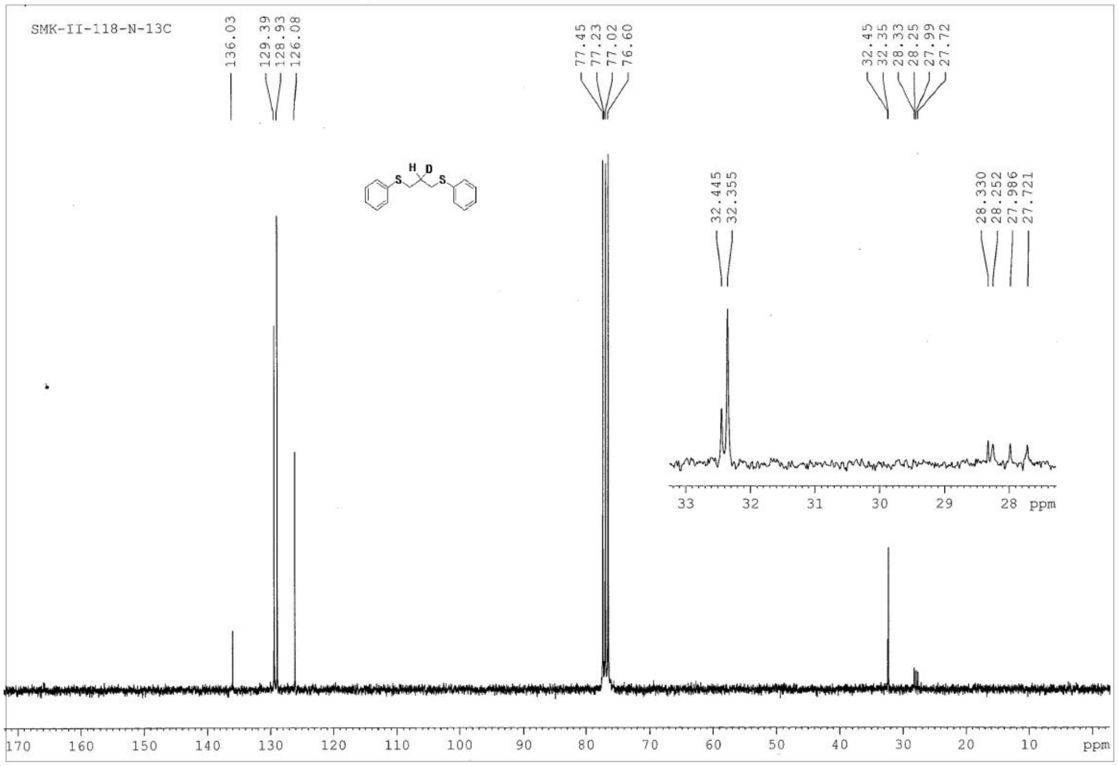

Supplement: File 2 — 1H NMR and 13C NMR spectra for compounds listed in Table 2, entries 1–19. [file Beilstein_J_Org_Chem-10-26-s002.pdf]
